# Supplementary material for: A Statistical Framework for Analysis of Trial-Level Temporal Dynamics in Fiber Photometry Experiments
Source: bioRxiv. 2024 Oct 19:2023.11.06.565896. Originally published 2023 Nov 6. Preprint. [Version 4] doi: 10.1101/2023.11.06.565896 (PMC10659337; doi:10.1101/2023.11.06.565896)
Supplement: 1 [file NIHPP2023.11.06.565896V4-supplement-1.pdf]

bioRxiv preprint doi: https://doi.org/10.1101/2023.11.06.565896; this version posted October 19, 2024. The copyright holder for this preprint (which was not certified by peer review) is the author/funder. This article is a US Government work. It is not subject to copyright under 17 USC 105 and is also made available for use under a CC0 license.

1 Appendix: Photometry Citations

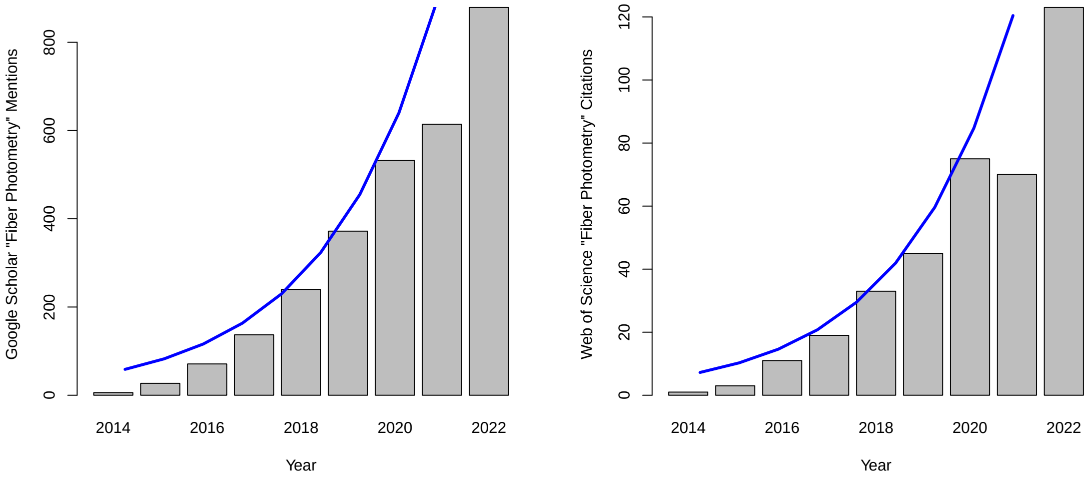

Figure 9: [Left] Google scholar mentions of the string "Fiber Photometry" by year. There were 549 mentions between January 1, 2023 – June 22, 2023. [Right] Web of Science citations of papers that include the string "Fiber Photometry" by year. There were 50 citations between January 1, 2023 – June 22, 2023. Blue lines indicate fitted values from an exponential fit to the data: $\texttt{Citations}_i = \alpha * \exp[\beta * \texttt{Year}_i]$, where α and β were estimated with the `nls` package in `R`. The 1,500 references to photometry described in the main text refers to Google Scholar mentions in the 12 months prior to June 2023.

2 Appendix: BH Correction

The goal of this appendix is to illustrate the advantages of *FLMM* compared to fitting LMMs to each trial time-point separately, and then applying a multiple-comparisons correction (as proposed in Lee et al. (2019)). We specifically show that, in contrast to the approach applied in that work, *FLMM* yields far less conservative and more stable inference across different sub-sampling rates. We analyzed the Delay Length experiment (shown in Figure 6) data sub-sampled at a range of sampling rates (data were sub-sampled at evenly spaced intervals). We fit either a collection of separate LMMs followed by a Benjamini–Hochberg (BH) correction, or *FLMM* with statistical significance determined from both Pointwise and Joint 95% CIs. To avoid introducing more notation, we show the *FLMM* model, with the understanding that the functional notation can be interpreted pointwise for the approach applied in Lee et al. (2019):

$$\mathbb{E}[Y_{i,j,l}(s) \,|\, \mathbf{X}_{i,l}, \mathbf{Z}_{i,l}, \boldsymbol{\gamma}_i(s)] = \beta_0(s) + \gamma_{0,i}(s) + \texttt{Delay}_{i,l}\left[\beta_1(s) + \gamma_{1,i}(s)\right].$$

As shown in Appendix Tables 1-2, the proportion of time-points, s, at which $\widehat{\beta}_0(s)$ is statistically significant with *FLMM* Joint CIs is fairly stable across sampling rates. In contrast, the percentage is highly inconsistent with the BH approach. For example, the BH approach identifies $\sim 30\%$ of time-points as significant at a sampling rate of 20Hz, less than 1% at 25Hz, $\sim 30\%$ at 30Hz and then drops again to $\sim 1\%$ at 40Hz. As the

bioRxiv preprint doi: https://doi.org/10.1101/2023.11.06.565896; this version posted October 19, 2024. The copyright holder for this preprint (which was not certified by peer review) is the author/funder. This article is a US Government work. It is not subject to copyright under 17 USC 105 and is also made available for use under a CC0 license.

sampling rate grows towards 125Hz (the sampling rate of the online dataset), the number of statistical comparisons grows, and the proportion of points that are significant drops to below 1%.

For the $\widehat{\beta}_1(s)$, the BH approach identifies no time-points that achieve statistical significance, even for time-points when the animal is consuming the reward. In contrast, the Pointwise and Joint *FLMM* CIs identify a relatively stable proportion of time-points that are statistically significant. The $\widehat{\beta}_1(s)$ is arguably a far more important point of comparison between these two statistical approaches, as the scientific question that motivated this analysis focuses on $\widehat{\beta}_1(s)$, rather than on $\widehat{\beta}_0(s)$.

Qualitatively speaking, the coefficient estimates, the widths of the 95% CIs, and the time intervals of statistical significance appear stable across the sampling rates, emphasizing how *FLMM* yields consistent inference and is not overly sensitive to the sub-sampling rate. We note that a multiple comparisons correction may yield more stable results if one first smooths regression coefficient point and variance estimates. To the best of our understanding, such smoothing was not conducted in Lee et al. (2019). Moreover, such a strategy would essentially be a functional mixed model using a multiple comparisons correction, instead of a joint CI.

**Sampling Rate (Hz)**	BH	Pointwise	Joint
**20**	31.00	23.58	9.17
**25**	0.73	23.27	9.09
**30**	30.23	22.67	8.72
**40**	1.09	22.66	8.50
**50**	0.73	22.38	8.28
**125**	0.80	22.02	7.63

Table 1: Functional intercept $\widehat{\beta}_0(s)$

**Sampling Rate (Hz)**	BH	Pointwise	Joint
**20**	0	46.72	17.90
**25**	0	44.36	16.73
**30**	0	43.31	15.41
**40**	0	43.57	15.90
**50**	0	43.46	14.39
**125**	0	42.15	13.66

Table 2: Functional slope $\widehat{\beta}_1(s)$

Tables 1-2: Percentage of time-points in which the coefficient estimates ($\widehat{\beta}_0(s)$, or $\widehat{\beta}_1(s)$) are statistically significant. We compare the Benjamini–Hochberg (BH) correction applied to pointwise LMM models, the *FLMM* pointwise 95% CIs (Pointwise), and the *FLMM* joint 95% CIs (Joint). [Left] The proportion of points that are significant with the BH approach jump around between 20-40Hz and then dramatically decrease as the sampling rate increases. In contrast, the *FLMM* Pointwise and Joint CIs are relatively stable and show only slight reductions in the proportion of points that are significant as the sampling rate increases. [Right] The BH identifies no statistically significant effects at any timespoints, whereas the the *FLMM* Pointwise and Joint CIs identify significant effects at a relatively consistent proportion of time-points.

3 Appendix: Functional Mixed Models Methods

For a thorough introduction to the Functional Mixed Model fitting framework presented in our manuscript, please refer to Cui et al. (2021). However, for completeness, we provide a short description of the estimation details and then provide a brief derivation of our proposed estimator. The majority of the details presented here can be found in greater depth in section *3.1. Analytic Inference for Gaussian Functional Data* of Cui

bioRxiv preprint doi: https://doi.org/10.1101/2023.11.06.565896; this version posted October 19, 2024. The copyright holder for this preprint (which was not certified by peer review) is the author/funder. This article is a US Government work. It is not subject to copyright under 17 USC 105 and is also made available for use under a CC0 license.

et al. (2021) but we believe we have included sufficient description of the statistical estimation scheme to explain the derivation of our proposed estimator (presented in Section 3.2). For clarity, we focus on functional *linear* mixed models. However, our package also provides the capability to fit the wider class of functional generalized linear mixed models for the distributions supported by the `R` `lme4` package.

3.1 Functional Linear Mixed Models

We focus on the functional linear mixed model,

$$\mathbf{Y}_i(s) = \mathbb{X}_i\boldsymbol{\beta}(s) + \mathbb{Z}_i\boldsymbol{\gamma}_i(s) + \boldsymbol{\epsilon}_i(s) \tag{4}$$

where $\boldsymbol{Y}_i(s) \in \mathbb{R}^{J_i}$ is the vector of observations of functional outcomes at time-point s for subject i, J_i is the number of functional observations of subject i, and $s \in \{1, 2, ..., S\}$, where S is the number of points on the grid of the functional domain (i.e., the number of trial time-points or trial photometry samples). We take n to be the number of subjects (e.g., animals) and $N = \sum_{i=1}^{n} J_i$. We denote $\boldsymbol{\beta}(s) \in \mathbb{R}^p$ to be the vector of functional fixed-effects at point s. $\boldsymbol{\gamma}_i(s) \in \mathbb{R}^q$ and $\boldsymbol{\epsilon}_i(s) \in \mathbb{R}^{J_i}$ are vectors of mutually independent random-effects and error terms for time-point s, with multivariate Gaussian distributions. We denote $\mathbb{X}_i \in \mathbb{R}^{J_i \times p}$ and $\mathbb{Z}_i \in \mathbb{R}^{J_i \times q}$ as (design) matrices of subject i containing the covariates for the fixed- and random-effects terms, respectively. Denote $\mathbb{Z} \in \mathbb{R}^{N \times nq}$ to be a block diagonal matrix where the ith block entry is $\mathbb{Z}_i$ and let $\mathbb{X} \in \mathbb{R}^{N \times p}$ refer to the design matrix from row concatenating the subject-specific matrices $\mathbb{X}_i$. Estimates for the fixed-effects $\boldsymbol{\beta}(s)$ are correlated across trial time-points (i.e., the functional domain), which is incorporated into *joint* inference by assuming $\text{Cov}\left(\boldsymbol{\gamma}(s_1), \boldsymbol{\gamma}(s_2)\right) = \mathbb{G}(s_1, s_2) \in \mathbb{R}^{nq \times nq}$ and $\text{Cov}\left(\boldsymbol{\epsilon}(s_1), \boldsymbol{\epsilon}(s_2)\right) = 0$ for all $s_1 \neq s_2$, where $\boldsymbol{\gamma}(s) = [\boldsymbol{\gamma}_1^T(s)\ \boldsymbol{\gamma}_2^T(s)\ ...\ \boldsymbol{\gamma}_n^T(s)]^T \in \mathbb{R}^{nq}$ and $\boldsymbol{\epsilon}(s) = [\boldsymbol{\epsilon}_1^T(s)\ \boldsymbol{\epsilon}_2^T(s)\ ...\ \boldsymbol{\epsilon}_n^T(s)]^T \in \mathbb{R}^N$.

We used a two-stage approach for fitting functional linear mixed models. In the first step, we fit pointwise linear mixed models at each time-point s, yielding fixed-effect estimates which we denote as $\tilde{\boldsymbol{\beta}}(s)$. The pointwise estimator admits the closed form expression, $\tilde{\boldsymbol{\beta}}(s) = \left[\mathbb{X}^T\mathbb{V}^{-1}(s)\mathbb{X}\right]^{-1}\mathbb{X}^T\mathbb{V}^{-1}(s)\boldsymbol{Y}(s)$, where $\mathbb{V}(s) = \mathbb{Z}\mathbb{H}(s)\mathbb{Z}^T + \tilde{\mathbb{R}}(s)$, and $\mathbb{H}(s)$ and $\tilde{\mathbb{R}}(s)$ are the covariance matrices of $\boldsymbol{\gamma}(s)$ and $\boldsymbol{\epsilon}(s)$, respectively. The variance of the pointwise estimator is $\text{Var}\left(\tilde{\boldsymbol{\beta}}(s)\right) = \left[\mathbb{X}^T\mathbb{V}^{-1}(s)\mathbb{X}\right]^{-1}$. The correlation across trial time-points can be seen more explicitly through the following expression

$$\text{Cov}\left(\tilde{\boldsymbol{\beta}}(s_1), \tilde{\boldsymbol{\beta}}(s_2)\right) = \left[\mathbb{X}^T\mathbb{V}^{-1}(s_1)\mathbb{X}\right]^{-1}\mathbb{X}^T\mathbb{V}^{-1}(s_1)\mathbb{W}(s_1, s_2)\mathbb{V}^{-1}(s_2)\mathbb{X}\left[\mathbb{X}^T\mathbb{V}^{-1}(s_2)\mathbb{X}\right]^{-1}$$

where $\mathbb{W}(s_1, s_2) = \mathbb{Z}\mathbb{G}(s_1, s_2)\mathbb{Z}^T$. While estimates of $\mathbb{H}(s)$ and $\tilde{\mathbb{R}}(s)$ are provided by standard mixed modeling software, $\mathbb{G}(s_1, s_2)$ must be estimated separately. We detail the estimation scheme below as it plays a critical role in *joint* inference and constitutes the main topic of our statistical contribution. Note that the estimates of $\mathbb{G}(s_1, s_2)$, $\mathbb{H}(s)$, and $\tilde{\mathbb{R}}(s)$ can be smoothed using, for example, fast bivaraite P-splines (Xiao et al., 2013) along the functional domain (that is, trial time-points) to reduce variability; and then any negative eigenvalues of the smoothed matrices can be trimmed at 0 to ensure the resulting covariance matrix estimates are positive semi-definite (Cui et al., 2021; Greven et al., 2010).

bioRxiv preprint doi: https://doi.org/10.1101/2023.11.06.565896; this version posted October 19, 2024. The copyright holder for this preprint (which was not certified by peer review) is the author/funder. This article is a US Government work. It is not subject to copyright under 17 USC 105 and is also made available for use under a CC0 license.

Our package provides the option to use an array of smoothing approaches for the regression coefficient estimates, $\tilde{\boldsymbol{\beta}}(s)$, but defaults to using penalized splines (Ruppert et al., 2003). When using penalized splines to smooth the pointwise estimates, $\tilde{\boldsymbol{\beta}}(s)$, the covariance matrix for the final fixed-effects estimates admits a closed form expression, and allows for fast calculation of *joint* confidence band estimates. To see this, denote $\widehat{\boldsymbol{\beta}}(s)$ as the final fixed-effect estimates after smoothing the raw pointwise estimates, $\tilde{\boldsymbol{\beta}}(s)$, directly provided by mixed model software. Then a smoothed estimator for the tth fixed-effect is $\widehat{\boldsymbol{\beta}}^{(t)} = \mathbb{S}_t\tilde{\boldsymbol{\beta}}^{(t)}$, where $\tilde{\boldsymbol{\beta}}^{(t)} = \left[\tilde{\beta}^{(t)}(s_1), ..., \tilde{\beta}^{(t)}(s_S)\right]^T \in \mathbb{R}^S$. Thus, $\text{Cov}\left(\widehat{\boldsymbol{\beta}}^{(t)}\right) = \mathbb{S}_t\text{Cov}\left(\tilde{\boldsymbol{\beta}}^{(t)}\right)\mathbb{S}_t$, where $\mathbb{S}_t = \mathbb{B}_t(\mathbb{B}_t^T\mathbb{B}_t + \lambda_t\mathbb{P}_t)^{-1}\mathbb{B}_t^T$, $\mathbb{B}_t$ is a K-dimensional spline basis matrix, λ_t is a smoothing parameter, and $\mathbb{P}_t$ is a penalty matrix. Typically the number of knots $K \ll S$, but as long as a sufficient number of knots are specified, the smoother and exact K selected appear to have little impact on the final coefficient estimates or 95% CIs in practice. To be conservative, we used a high number of knots ($K = S/4$). In practice, the specific number could be altered depending on the sampling rate of the photometry data analyzed and the specific sensor (since the kinetics of neurochemical signaling systems can vary widely). The results presented in the paper were calculated based upon implementation of our method using thin-plate splines since these performed well in practice.

Our package leverages the well-known `lme4` package in `R` for fitting the pointwise mixed effects models (Bates, 2010; Bates et al., 2014). This precludes, however, the specification of structure on the covariance matrix of the errors, $\boldsymbol{\epsilon}_i(s)$ across trials (for a fixed trial time-point s) in Gaussian models, functionality provided by, for example, the `nlme` in `R` (Pinheiro et al., 2023). Future work could explore functional linear mixed models that allow for this functionality. However, in our experimentation analyzing photometry data summary measures (e.g., AUCs) from a range of experiments that observed multiple trials and sessions per animal, we found that model fit criteria (e.g., AIC, BIC, cAIC (Säfken et al., 2018)) were usually much better when accounting for correlation across trials within-animal through random-effect specifications as opposed to placing structure on the covariance of the errors within-subject across trials. Moreover, we found that models that specified the types of error covariance structures applicable to photometry experiments were often slow to fit which is a drawback in our approach to fitting functional mixed models given that it requires fitting many pointwise models. For those reasons, we set our package's default to use the `lme4` package thereby providing both speed and modeling flexibility to users.

We now provide a brief description of our proposed estimator for $\mathbb{G}(s_1, s_2)$. The critical role that this plays in inference and in neuroscience applications arises from the nested designs common in the sophisticated behavioral experiments commonly used alongside fiber photometry. We briefly describe this in Appendix Section 3.3.

3.2 Appendix: Covariance Estimator

We begin with a high-level description of the estimator for the covariance matrix, $\mathbb{G}(s_1, s_2)$, and include details below. Denote $Y_{i,j}(s_1) \in \mathbb{R}$ as the functional outcome at time-point s_1, for subject i on trial j. The method of moments estimator proposed in (Greven et al., 2010) and applied to our functional mixed model estimation procedure

bioRxiv preprint doi: https://doi.org/10.1101/2023.11.06.565896; this version posted October 19, 2024. The copyright holder for this preprint (which was not certified by peer review) is the author/funder. This article is a US Government work. It is not subject to copyright under 17 USC 105 and is also made available for use under a CC0 license.

was presented in Section 3.1, equation (4) from Cui et al. (2021),

$$\mathbb{E}\left[\{Y_{i,k}(s_1) - \mathbf{X}_{i,k}^T\boldsymbol{\beta}(s_1)\}\{Y_{i,j}(s_2) - \mathbf{X}_{i,j}^T\boldsymbol{\beta}(s_2)\}\right] = \sum_{t=1}^{q}\sum_{v=1}^{q} Z_{i,k,t}Z_{i,j,v}\text{Cov}\left(\gamma_{i,t}(s_1), \gamma_{i,v}(s_2)\right) \quad (5)$$

where t, v are random-effect covariate indices. This expression suggests an estimator that regresses the residual products $[Y_{i,k}(s_1) - \mathbf{X}_{i,k}^T\boldsymbol{\beta}(s_1)][Y_{i,j}(s_2) - \mathbf{X}_{i,j}^T\boldsymbol{\beta}(s_2)]$ onto the random-effect covariate products $\{Z_{i,k,t}Z_{i,j,v} \ : \ j, k = 1, 2, ...J_i\}$. Concatenating these covariate products into a design matrix, $\tilde{\mathbb{Z}}$, and concatenating the residual products into an "outcome vector", $\tilde{\mathbf{Y}}(s_1, s_2) \in \mathbb{R}^N$, allows us to express the estimator as a solution to the least squares problem for each pair (s_1, s_2)

$$\widehat{\boldsymbol{\alpha}}(s_1, s_2) \in \underset{\boldsymbol{\alpha}(s_1,s_2)}{\text{argmin}} \left\|\tilde{\mathbf{Y}}(s_1, s_2) - \tilde{\mathbb{Z}}\boldsymbol{\alpha}(s_1, s_2)\right\|_2^2 \quad (6)$$

where $\tilde{\mathbb{Z}} \in \mathbb{R}^{N\times\tilde{q}}$ is a matrix of the covariate products, $Z_{i,k,t}Z_{i,j,v}$, described above. After calculating $\widehat{\boldsymbol{\alpha}}(s_1, s_2)$, $\widehat{\mathbb{G}}(s_1, s_2)$ is obtained by re-organizing the elements of the vector $\widehat{\boldsymbol{\alpha}}(s_1, s_2)$ into the entries of the matrix $\widehat{\mathbb{G}}(s_1, s_2)$. This estimator is flexible and in principle is agnostic to the random-effect specification, but in practice requires extension for general random-effects specifications, which we derive here.

Before discussing the extension to this estimator, we provide additional necessary notation. Since a single random-effect can require many columns of $\mathbb{Z}$ to encode the corresponding covariate (e.g., if there are many session-specific random intercepts), separate indices are needed to distinguish between a single random-effect distribution and the (potentially many) columns of the random-effect design matrix associated with draws from that random-effect distribution.

The $\tilde{\mathbb{Z}} \in \mathbb{R}^{N\times\tilde{q}}$, described above, is a block diagonal matrix where the ith block entry is $\tilde{\mathbb{Z}}_i$. The number of columns, $\tilde{q}$, will vary depending on the random-effect specification. For example, if one specifies a model that includes session-specific random-intercepts, this would require one column in $\mathbb{Z}$ (and thus multiple columns in $\tilde{\mathbb{Z}}$) for each animal and session to encode the corresponding animal- and session-specific covariates associated with these random-intercepts.

Let q^* be the number of unique random-effects distributions for a given model, where $q^* \leq q$. Take as an example a model that includes: 1) a subject-specific random intercept, $\gamma_{0,i}(s)$, and 2) subject- and session-specific random intercepts, $\gamma_{1,i,l}(s)$ for participant i on session l. If we assume that $\gamma_{1,i,l}(s) \overset{iid}{\sim} \mathcal{N}(0, \tilde{\sigma}^2) \ \forall \ i, l$, then $q^* = 2$, but $q > 2$ (and thus $\tilde{q} > 2$) since encoding the subject- and session-specific random intercepts, $\gamma_{1,i,l}(s)$ would require many indicator variables (entered as columns of $\mathbb{Z}_i$). More generally, for random-effect covariate, r, denote $\mathcal{I}_r$ as the set of column indices of $\mathbb{Z}$ that encode random-effect covariate r. Although we describe $\gamma_{r,i,l}(s)$ as if it were drawn from a univariate Gaussian for explanatory purposes, neither the estimator nor the modeling software assume independence between random-effects from *different* covariates.

Expanding Section 3.1, equation (4) from Cui et al. (2021) to be in terms of the

bioRxiv preprint doi: https://doi.org/10.1101/2023.11.06.565896; this version posted October 19, 2024. The copyright holder for this preprint (which was not certified by peer review) is the author/funder. This article is a US Government work. It is not subject to copyright under 17 USC 105 and is also made available for use under a CC0 license.

column indices of the design matrix $\mathbb{Z}_i$,

$$\begin{aligned}
&\sum_{t=1}^{q}\sum_{v=1}^{q} Z_{i,k,t}Z_{i,j,v}\text{Cov}\left(\gamma_{i,t}(s_1),\gamma_{i,v}(s_2)\right)\\
&=\sum_{r=1}^{q^*}\sum_{m=1}^{q^*}\sum_{w\in\mathcal{I}_r}\sum_{b\in\mathcal{I}_m} Z_{i,k,w}Z_{i,j,b}\text{Cov}\left(\gamma_{i,w}(s_1),\gamma_{i,b}(s_2)\right)\\
&=\sum_{r=1}^{q^*}\sum_{m=1}^{q^*}\left\{\sum_{w\in\mathcal{I}_r} Z_{i,k,w}\left[\sum_{b\in\mathcal{I}_m} Z_{i,j,b}\text{Cov}\left(\gamma_{i,w}(s_1),\gamma_{i,b}(s_2)\right)\right]\right\}.
\end{aligned}$$

Now recall that $\gamma_{r,i,w}(s) \overset{iid}{\sim} \mathcal{N}(0,\tilde{\sigma}_r^2)$ for all $w \in \mathcal{I}_r$. Thus, $\text{Cov}\left(\gamma_{r,i,w}(s_1),\gamma_{r,i,b}(s_2)\right) = \tilde{\rho}_{r,m}$ for a covariance value, $\tilde{\rho}_{r,m}$, that is equal across all $w \in \mathcal{I}_r$ and $b \in \mathcal{I}_m$. It then follows that we can simplify the above as,

$$\begin{aligned}
&\sum_{r=1}^{q^*}\sum_{m=1}^{q^*}\left\{\sum_{l\in\mathcal{I}_r} Z_{i,k,l}\left[\sum_{b\in\mathcal{I}_m} Z_{i,j,b}\text{Cov}\left(\gamma_{i,l}(s_1),\gamma_{i,b}(s_2)\right)\right]\right\}\\
&=\sum_{r=1}^{q^*}\sum_{m=1}^{q^*}\left[\sum_{l\in\mathcal{I}_r} Z_{i,k,l}\right]\left[\sum_{b\in\mathcal{I}_m} Z_{i,j,b}\right]\text{Cov}\left(\gamma_{i,r}(s_1),\gamma_{i,m}(s_2)\right).
\end{aligned}$$

This expression suggests an estimation strategy that takes the product $\left[\sum_{l\in\mathcal{I}_r} Z_{i,k,l}\right]\left[\sum_{b\in\mathcal{I}_m} Z_{i,j,b}\right]$ as a "covariate" in the OLS-based estimator described in expression (5). Organizing these products into the columns of $\tilde{\mathbb{Z}}$ does not result, however, in a full rank matrix for all random-effect specifications. In such cases, the solution to problem (6) is not unique. We solve for the "ridgeless regression" solution to problem (6) because it yields the minimum ℓ_2 norm estimator and exhibits desirable statistical properties (Hastie et al., 2019). This can be expressed as the solution to the optimization problem,

$$\begin{aligned}
&\min_{\boldsymbol{\alpha}(s_1,s_2)} \|\boldsymbol{\alpha}(s_1,s_2)\|_2^2 \\
&\text{s.t.} \quad \boldsymbol{\alpha}(s_1,s_2) = \underset{\boldsymbol{\alpha}(s_1,s_2)}{\text{argmin}} \left\|\widehat{\boldsymbol{\epsilon}}(s_1,s_2) - \tilde{\mathbb{Z}}\boldsymbol{\alpha}(s_1,s_2)\right\|_2^2
\end{aligned} \tag{7}$$

where $\widehat{\boldsymbol{\epsilon}}(s_1,s_2)$ are the residual products (i.e., with entries $\widehat{\epsilon}_{i,j,k}(s_1,s_2) = [Y_{i,k}(s_1) - \mathbf{X}_{i,k}^T\boldsymbol{\beta}(s_1)][Y_{i,j}(s_2) - \mathbf{X}_{i,j}^T\boldsymbol{\beta}(s_2)]$) and $\boldsymbol{\alpha}(s_1,s_2) \in \mathbb{R}^{\tilde{q}}$. We construct $\widehat{\mathbb{G}}(s_1,s_2)$ through reorganizing the elements of $\widehat{\boldsymbol{\alpha}}(s_1,s_2)$ into the matrix $\widehat{\mathbb{G}}(s_1,s_2)$. The $\boldsymbol{\alpha}(s_1,s_2)$ can be estimated with the closed form expression,

$$\widehat{\boldsymbol{\alpha}}(s_1,s_2) = \left(\tilde{\mathbb{Z}}^T\tilde{\mathbb{Z}}\right)^+ \tilde{\mathbb{Z}}^T\tilde{\mathbf{Y}}(s_1,s_2) = \mathbb{M}\tilde{\mathbf{Y}}(s_1,s_2),$$

where $(\cdot)^+$ denotes the Moore-Penrose pseudoinverse inverse (Hastie et al., 2019). Thus,

bioRxiv preprint doi: https://doi.org/10.1101/2023.11.06.565896; this version posted October 19, 2024. The copyright holder for this preprint (which was not certified by peer review) is the author/funder. This article is a US Government work. It is not subject to copyright under 17 USC 105 and is also made available for use under a CC0 license.

while the estimator requires one to solve $S(S+1)/2$ problems of the form above (i.e., one for each unique $\{s_1, s_2\}$ pair), in practice we only need to calculate $\mathbb{M}$ once for all $\{s_1, s_2\}$ pairs. This allows for estimation of $\mathbb{G}$ (i.e., for all unique $\{s_1, s_2\}$ pairs) for datasets with many observations, complex random-effects specifications, and large S within a couple seconds in total. We found that this approach performed well in practice, and it is the default in our package. All results presented in the main text apply to this version.

The above estimator allows for a fast, flexible extension of the work in (Cui et al., 2021), thereby allowing for 95% CI calculation for general random-effects specifications. This statistical contribution is critical for neuroscience, since studies often exhibit nested experimental designs that require sophisticated random-effect models to properly capture the rich information contained in photometry signals.

3.2.1 Alternative Covariance Estimators

We also explored a collection of related strategies. These are motivated by the fact that one can regress the residual products onto $\{Z_{i,k,v}Z_{i,j,t} \;:\; v, t = 1, ..., q\}$, instead of onto the products of the sums (across indices t, v), as proposed above. Denote $\widehat{\alpha}_{v,t}$ as the coefficient estimate associated with the "covariate" $Z_{i,j,v}Z_{i,k,t}$ in this regression. We exploit the fact that $\text{Cov}\left(\gamma_{i,w}(s_1), \gamma_{i,b}(s_2)\right) = \tilde{\rho}_{r,m}$ for a covariance value, $\tilde{\rho}_{r,m}$, common across all $w \in \mathcal{I}_r$ and $b \in \mathcal{I}_m$. Solving for the desired quantity then yields the estimator

$$\widehat{\text{Cov}}\left(\gamma_{i,r}(s_1), \gamma_{i,m}(s_2)\right) = \frac{1}{|\mathcal{I}_r||\mathcal{I}_m|} \sum_{w \in \mathcal{I}_r} \sum_{b \in \mathcal{I}_m} \widehat{\alpha}_{w,b}. \tag{8}$$

We found this approach (available in our package by setting the argument `MoM = 2`) was slower, more memory intensive, and yielded confidence interval coverage comparable to the approach proposed above. We also explored the performance of two estimators for $\mathbb{G}$ based on mathematical programs that enforce non-negativity of variance components (before eigenvalue trimming). The first mathematical program is

$$\begin{aligned} &\min_{\boldsymbol{\alpha}(s_1,s_2)} \|\boldsymbol{\alpha}(s_1, s_2)\|_2^2 \\ &\text{s.t.} \quad \boldsymbol{\alpha}(s_1, s_2) = \underset{\boldsymbol{\alpha}(s_1,s_2)}{\text{argmin}} \left\|\widehat{\boldsymbol{\epsilon}} - \tilde{\mathbb{Z}}\boldsymbol{\alpha}(s_1, s_2)\right\|_2^2 \\ &\alpha_m \geq 0 \quad \text{for } m \in \mathcal{M} \end{aligned} \tag{9}$$

where $\mathcal{M}$ denotes the set of indices corresponding to variance elements.

Expression (8) suggests that one could alternatively constrain the sum, as opposed to each element in the summation $\sum_{l \in \mathcal{I}_r} \sum_{b \in \mathcal{I}_m} \widehat{\alpha}_{l,b}$. Thus we also explored performance

bioRxiv preprint doi: https://doi.org/10.1101/2023.11.06.565896; this version posted October 19, 2024. The copyright holder for this preprint (which was not certified by peer review) is the author/funder. This article is a US Government work. It is not subject to copyright under 17 USC 105 and is also made available for use under a CC0 license.

of the estimator based on the mathematical program,

$$\begin{aligned} &\min_{\boldsymbol{\alpha}(s_1,s_2)} \|\boldsymbol{\alpha}(s_1,s_2)\|_2^2 \\ &\text{s.t.} \quad \boldsymbol{\alpha}(s_1,s_2) = \underset{\boldsymbol{\alpha}(s_1,s_2)}{\operatorname{argmin}} \left\|\widehat{\boldsymbol{\epsilon}} - \tilde{\mathbb{Z}}\boldsymbol{\alpha}(s_1,s_2)\right\|_2^2 \\ &\sum_{l \in \mathcal{I}_m} \alpha_l \geq 0 \quad \text{for } m \in \mathcal{M}. \end{aligned} \tag{10}$$

We found that, in practice, the performance of the estimators based on the mathematical programs (7), (9), and (10), performed comparably in simulations. This may be because the eigenvalue trimming applied to the solutions to the above optimization problems yields similar final estimates. Our software automatically implements estimators based on all approaches described above, but defaults to the first estimator proposed above, which is based on the solution to mathematical program (7).

In simulations, we observed that the regression coefficient estimate 95% CIs calculated using our proposed $\mathbb{G}$ estimator achieved nearly nominal *joint* coverage even with model specifications that yield reduced rank $\tilde{\mathbb{Z}}$. Therefore, the above estimator allows for a fast, flexible extension of the work in (Cui et al., 2021), thereby allowing for 95% CI calculation for general random-effects specifications. This statistical contribution is important for neuroscience since studies often exhibit nested experimental designs that require sophisticated random-effect models to properly capture the rich information contained in photometry signals.

3.2.2 Reduced Rank Problem

We point out that the method of moments estimators applied here (proposed in Greven et al. (2010)) exhibit a non-identifiability property. While this did not substantially influence the resulting 95% confidence interval coverage in our simulations, we argue that future attention to this issue is warranted. Such settings arise anytime one includes, for example, nested random-effect structures. This is a drawback because these types of random-effect specifications are critical to properly model the sophisticated behavioral designs common in neuroscience which often exhibit multiple layers of nesting. For instance, suppose a collection of animals are trained on multiple sessions, and within each session are trained on multiple trials. Then trial is nested in session, which is nested in animal/subject. Inclusion of animal- and session-specific random intercepts, a common random-effect specification for such experimental designs, will exhibit the challenges above.

In order to make the issue concrete, we describe perhaps the simplest case where the above problem presents. Specifically, we show how the original estimator requires extension in a simple *FLMM* model that is analogous to the functional analogue of the paired samples t-test: a model that includes a single binary covariate such as a treatment indicator (i.e., $X_{i,j} \in \{0,1\}$), a random subject-specific intercept, and a random subject-specific slope. Suppose we observe a collection of trials indexed by j, on a collection of animals indexed by i. Then the above model can be expressed as

$$\mathbb{E}[Y_{i,j}(s) \mid \mathbf{X}_{i,j}, \mathbf{Z}_{i,j}, \boldsymbol{\gamma}_i(s)] = \beta_0(s) + \gamma_{0,i}(s) + X_{i,j}\left[\beta_1(s) + \gamma_{1,i}(s)\right]. \tag{11}$$

bioRxiv preprint doi: https://doi.org/10.1101/2023.11.06.565896; this version posted October 19, 2024. The copyright holder for this preprint (which was not certified by peer review) is the author/funder. This article is a US Government work. It is not subject to copyright under 17 USC 105 and is also made available for use under a CC0 license.

This model yields the vector of random-effect covariates $\mathbf{Z}_{i,j}^T = [1 \quad X_{i,j}]$, and the associated matrix $\tilde{\mathbb{Z}}_i$ is

$$\tilde{\mathbb{Z}}_i = \begin{bmatrix} 1 & 2X_{1,1} & X_{1,1}^2 \\ 1 & 2X_{1,2} & X_{1,2}^2 \\ \vdots & \vdots & \vdots \\ 1 & 2X_{1,J_1} & X_{1,J_1}^2 \end{bmatrix} = \begin{bmatrix} 1 & 2X_{1,1} & X_{1,1} \\ 1 & 2X_{1,2} & X_{1,2} \\ \vdots & \vdots & \vdots \\ 1 & 2X_{1,J_1} & X_{1,J_1} \end{bmatrix}.$$

The above equality follows trivially because $X_{i,j}^2 = X_{i,j}$ since $X_{i,j}$ is binary. The columns therefore exhibit the linear dependence $2\tilde{\mathbb{Z}}_{\cdot,2} = \tilde{\mathbb{Z}}_{\cdot,3}$ and thus $\tilde{\mathbb{Z}}$ is not full rank. To our knowledge, no re-coding of the binary variable $X_{i,j}$ resolves the above linear dependence problem without changing the contrast (e.g., one could fit a model without a random intercept and include the covariates $[X_{i,j} \quad (1 - X_{i,j})]$.

More generally, the reduced rank problem presents whenever a subject-specific random intercept is included (because it is encoded by a column of ones, $\mathbb{1}$, in $\tilde{\mathbb{Z}}$) alongside any binary covariate in $\mathbb{Z}$. As shown in the example above, this is because the column of ones used for random intercepts, when multiplied by binary covariates to construct the matrix $\tilde{\mathbb{Z}}$, will create linear dependence in the columns of $\tilde{\mathbb{Z}}$. This is problematic because binary covariates are needed to encode dichotomous and factor covariates (e.g., in the functional analogue of t-tests and ANOVAs), as well as, for example, session-specific random intercepts in nested designs. As such, resolving this issue is critical to properly model experiments that regularly arise in neuroscience.

3.3 Appendix: Random-Effects Structures and Interpretations for Neuroscience Experiments

Functional random intercepts that are unique to each trial and/or sessions can be used to account for animal-to-animal, session-to-session, and trial-to-trial heterogeneity in the signal and is one example where one might include a covariate in the random-effects (that is, included in $\mathbb{Z}_i$) but not in the main effects terms, $\mathbb{X}_i$. Inclusion of these session- and trial-level random-effects is an example of one way to account for correlation within an animal across trials and sessions (“photometry signal on trial j for animal i is likely similar to the signal on trial $j+1$). Indeed, many neuroscience studies involving behavioral paradigms that involve multiple sessions and trials (and potentially multiple groups) may require complicated random-effects structures to account for animal-to-animal, session-to-session and trial-to-trial heterogeneity in the photometry signal. For this reason, we explored the performance of many models to learn how to best model variability in the data.

FLMM yields interpretable regression coefficient estimates. Conveniently, they can be interpreted conditional on the functional random-effects or marginally with respect to the random-effects since both interpretations are numerically equivalent in the linear setting. For an intuitive introduction to this equivalence for linear mixed models, we recommend sections 2.2 and 7.4 of Fitzmaurice et al. (2008). We present the marginal interpretation here because it is often more intuitive for photometry analyses. However, the conditional version has its own advantages.

bioRxiv preprint doi: https://doi.org/10.1101/2023.11.06.565896; this version posted October 19, 2024. The copyright holder for this preprint (which was not certified by peer review) is the author/funder. This article is a US Government work. It is not subject to copyright under 17 USC 105 and is also made available for use under a CC0 license.

3.4 Functional Regression Model Classes

	**Longitudinal**	**Cross-Sectional**
**Functional**	Functional Mixed Models	Functional Regression
**Scalar**	Generalized Linear Mixed-Effects (GLMM)	Generalized Linear Models (GLM)

Table 3: Regression model classes based on functional vs. scalar response variables and for longitudinal (repeated measures) vs. cross-sectional data. We use "functional mixed models" as a short-hand for function-on-scalar mixed models. We use "functional regression" as a short-hand for single level function-on-scalar regression.

	**Functional Outcome**	**Scalar Outcome**
**Functional Predictors**	Function-on-Function Regression (FoFR)	Scalar-on-Function Regression (SoFR)
**Scalar Predictors**	Function-on-Scalar Regression (FoSR)	Generalized Linear Models (GLM)

Table 4: Cross-sectional regression model classes based on functional vs. scalar predictor variables (i.e., covariates) and functional vs. scalar outcome variables. We take the FoFR, FoSR and SoFR to be the single level (non-longitudinal) versions of these methods.

4 Appendix: Reanalysis Figures and Methods

In the following sections, we provide additional details for the reanalyses (Jeong et al., 2022) presented in the main text. In cases where it is helpful, we provide quotes from the original paper (Jeong et al., 2022) that informed our modeling decisions. These were taken from the main text of the paper, figure captions, and the supplement. In some places, we omitted minor portions of text to make the quotations more concise (e.g., figure references or citation numbers).

bioRxiv preprint doi: https://doi.org/10.1101/2023.11.06.565896; this version posted October 19, 2024. The copyright holder for this preprint (which was not certified by peer review) is the author/funder. This article is a US Government work. It is not subject to copyright under 17 USC 105 and is also made available for use under a CC0 license.

4.1 Section 2.3 Reanalyses

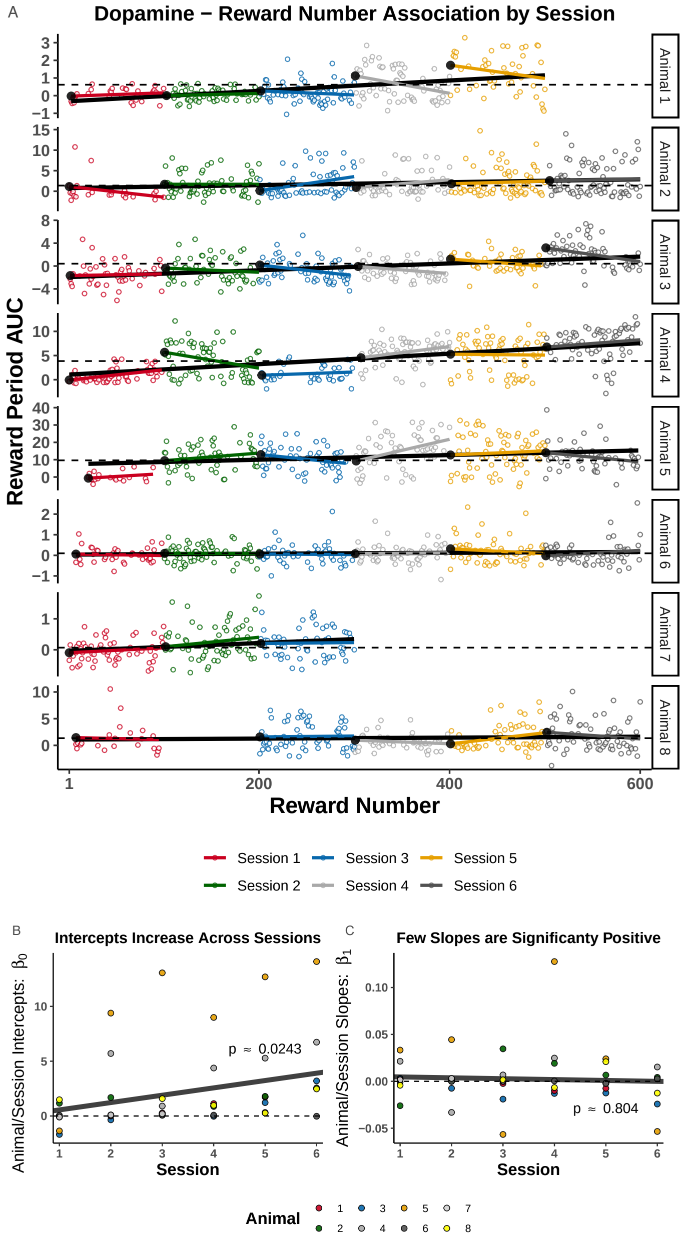

Figure 10: `Reward Number`–**AUC correlation within-session and across-session.** **(A)** Color indicates session number, rows denote animal number. `Trial Number` is the within-session `Reward Number` and ranges from 1-100 for each session. The black line that spans across sessions is a `Reward Number`–AUC linear regression fit, while the session color lines indicate a within-session `Trial Number`–AUC linear regression fit. The large black circles on the left side of each session-specific fit is the intercept, paramterized to yield the interpretation as the "expected AUC on the first trial of the corresponding session." Dotted horizontal lines are set at the median of the intercepts to facilitate comparison. The intercepts tend to rise across sessions, while few slopes are significantly positive. **(B)-(C)** Each dot indicates the estimated intercept value (B) or slope (C) from the fits shown in (A). Lines and p-values were calculated in an LMM that was fit to the session-specific linear regression slopes, $\widehat{\boldsymbol{\beta}}_1$, and intercepts, $\widehat{\boldsymbol{\beta}}_0$, shown in (A). The LMM included animal-specific random intercepts and slopes. These plots quantify the trend observed in (A): the estimated intercepts significantly increase across sessions, but the slopes are mostly negative.

bioRxiv preprint doi: https://doi.org/10.1101/2023.11.06.565896; this version posted October 19, 2024. The copyright holder for this preprint (which was not certified by peer review) is the author/funder. This article is a US Government work. It is not subject to copyright under 17 USC 105 and is also made available for use under a CC0 license.

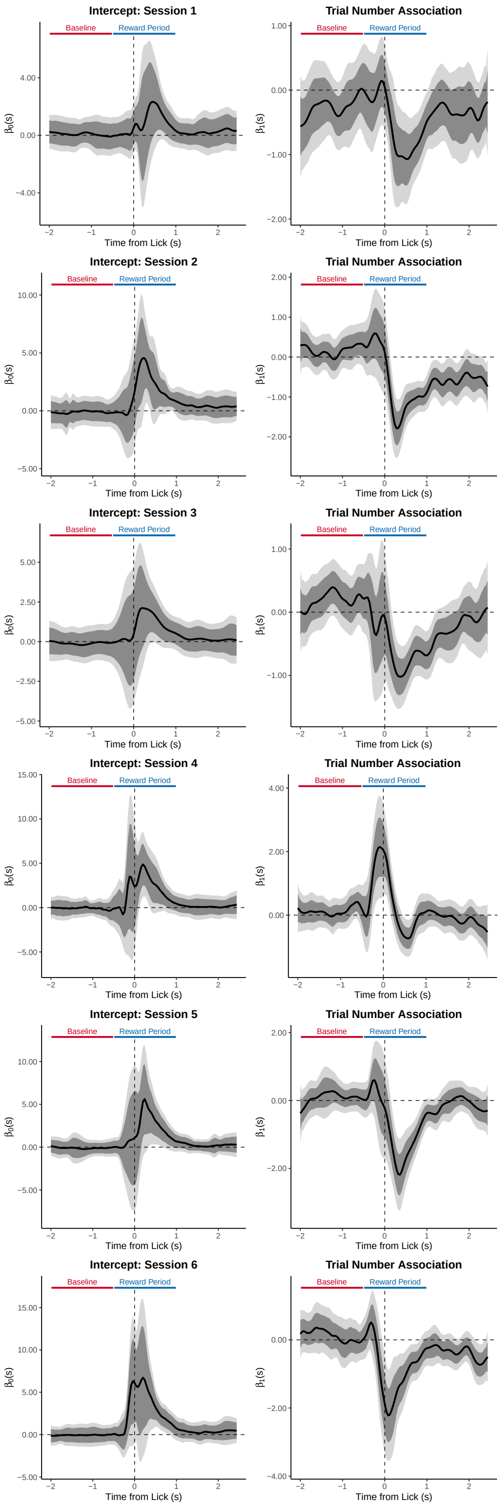

Figure 11: **`Trial Number`–DA correlation within-session on the random IRI task.** Row indicates session number. The intercept is paramterized to yield the interpretation as the "expected signal magnitude on the first trial of the corresponding session." Effects are aligned to the first lick after reward delivery. This is the session-by-session version of the analysis presented in Figure 5J-K.

bioRxiv preprint doi: https://doi.org/10.1101/2023.11.06.565896; this version posted October 19, 2024. The copyright holder for this preprint (which was not certified by peer review) is the author/funder. This article is a US Government work. It is not subject to copyright under 17 USC 105 and is also made available for use under a CC0 license.

4.2 Appendix: Section Lick Bout Correlation

We present further analyses that illustrate how *FLMM* reveals effects obscured by standard methods. The authors note that the `Reward Number`–DA association, reported in their analyses, could arise from `Lick Rate` increases that also correlate with `Reward Number`. They tested this alternative explanation by applying a Pearson correlation between `Lick Rate` and DA. While they reported no significant association, the plot for the `Lick Rate` covariate from *FLMM* (Figure 12), shows `Lick Rate` exhibits 1) a significant positive association with DA before Lick-bout onset, and 2) a negative association after Lick-bout onset (reaching *joint* significance at $\sim 1sec$). These results suggest that the correlation analysis of reward period AUC in the paper missed this effect because the AUC summarized a time-window that contained opposing effects. The underlying association was likely diluted by averaging over time-points when `Lick Rate` is both positively correlated with DA (the first 0.5 sec of the reward period, visible in positive *FLMM* coefficient estimates) and negatively correlated with DA (the final 1 sec of the 1.5 sec time-window, with negative *FLMM* coefficient estimates). Identifying this effect with a summary measure would have required selecting these time intervals perfectly a priori; this is not necessary with *FLMM*.

bioRxiv preprint doi: https://doi.org/10.1101/2023.11.06.565896; this version posted October 19, 2024. The copyright holder for this preprint (which was not certified by peer review) is the author/funder. This article is a US Government work. It is not subject to copyright under 17 USC 105 and is also made available for use under a CC0 license.

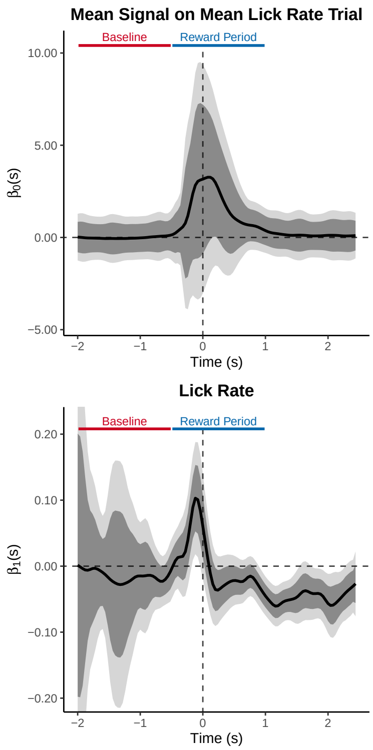

Figure 12: ***FLMM* reveals details occluded by summary measure analyses.** Coefficient estimates from an *FLMM* analysis of the random inter-trial interval (`IRI`) reward delivery experiment. The top row contains the intercept term plots where the title provides interpretation of the intercept: the average dopamine (DA) signal on trials when `Lick Rate` is at its average value. The bottom row shows the coefficient estimate plot of the covariate in the model. The "Baseline" and "Reward Period" bars show the trial period that the original authors used to calculate the summary measure (AUC). Specifically, they quantified DA by a measure of normalized AUC of $\Delta F/F$ during a window 0.5 s before to 1 sec after the first lick following reward delivery. All plots are aligned to this first lick after reward delivery. The interpretation of the y-value of the bottom plot at any time-point s: the mean change in the dopamine signal at s for a one unit change in `Lick Rate`. Association between DA and `Lick Rate` aligned to lick bout onset. Time-points when `Lick Rate` was negatively associated with DA (negative coefficient estimates in the final 1 sec of the 1.5 sec window) may have diluted time-points when they were positively associated (positive coefficient estimates in the first 0.5 sec of the reward period).

`Lick Rate` Model We conducted an *FLMM* reanalysis of the analyses shown in Figure S8 C-D of (Jeong et al., 2022). The figure caption notes that "The consummatory lick rate is not correlated with dopamine reward response." We used the methods described in the quoted paragraphs above, and provide further details in Appendix Section 4.2. We fit the lick rate ($\texttt{LR}_{i,j,l}$) *FLMM*

$$\begin{aligned}\mathbb{E}[Y_{i,j,l}(s) \mid \mathbf{X}_{i,j,l}, \mathbf{Z}_{i,j,l}, \boldsymbol{\gamma}_i(s)] = &\beta_0(s) + \beta_1(s)\texttt{LR}_{i,j,l} + \gamma_{0,i}(s) + \\ &\gamma_{1,i,l}(s) + \texttt{TN}_{i,j,l}\left[\gamma_{2,i}(s) + \gamma_{3,i,l}(s)\right]\end{aligned}$$

where `Trial Number` is denoted as $\texttt{TN}_{i,j,l}$. This can be fit with the code:

```
model_fit = fui(photometry ~ lick_rate + (trial | id/session),
                data = photometry_data,
                subj_ID = "id")
```

bioRxiv preprint doi: https://doi.org/10.1101/2023.11.06.565896; this version posted October 19, 2024. The copyright holder for this preprint (which was not certified by peer review) is the author/funder. This article is a US Government work. It is not subject to copyright under 17 USC 105 and is also made available for use under a CC0 license.

Consummatory Lick-bout extraction As stated in "Data Analysis: Experiment 1" of the Supplement (p.3-4), consummatory Lick-bouts were defined as follows:

> "To test whether lick rate affects dopamine reward response, we first classified licks into consummatory and non-consummatory licks. A group of licks with less than 1 s interval was defined as a lick bout...Every lick in the first lick bout after reward delivery was defined as a consummatory lick, and all other licks were defined as nonconsummatory licks...To avoid any influence of the previous reward on the dopamine response or behavior to the current reward, we excluded rewards with less than 3 s IRI from the previous reward ($22.6 \pm 2.4\%$) for the above analyses. Rewards without lick until the next reward ($5.7 \pm 0.1\%$) were also excluded from analyses."

For an IRI that was less than 3 sec, we excluded the trial before and after that IRI so as to avoid any influence on either trial's signal. Our code implementations of the above methods can be found on the github page: `https://github.com/gloewing/photometry_fGLMM`.

The reward period time-window and methods were described in the Supplement of Jeong et al. (2022):

> "Dopamine response to reward was defined as the normalized area under curve (AUC) of $\Delta F/F$ during reward period. Reward period was defined as -0.5 to 1 s from the first lick after reward delivery. We defined the window with reference to the first lick time, not reward delivery time, because the response latency to reward differs across trials. Also, we used a window starting from 0.5 s ahead the first lick because dopamine response started to increase even before an animal made the first lick (as they get better at sensing reward delivery in late sessions). The AUC during 1.5 s time window before reward period was subtracted from AUC during reward period to normalize baseline activity."

4.3 Photobleaching

In main text Section 2.3, we conducted analyses that showed DA decreased within-session during the post-lick interval of the reward period ([0, 1] sec from Lick-onset): `Trial Number` was negatively correlated with signal magnitude during the post-lick period. We include a portion of main Figure 5 here to assist in the photobleaching discussion.

bioRxiv preprint doi: https://doi.org/10.1101/2023.11.06.565896; this version posted October 19, 2024. The copyright holder for this preprint (which was not certified by peer review) is the author/funder. This article is a US Government work. It is not subject to copyright under 17 USC 105 and is also made available for use under a CC0 license.

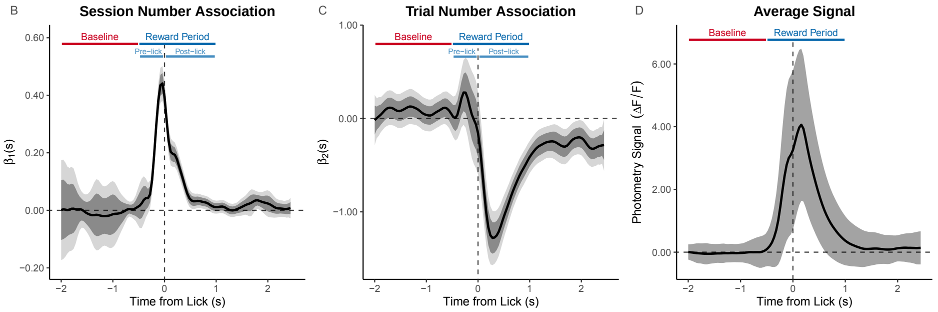

Figure 13: **Random `IRI` experiment aligned to Lick-onset: `Reward Number`–DA association analyzed as within-session (`Trial Number`) and between-session (`Session Number`) linear effects.** The average reward signal shows the average trace with standard error of the mean indicated by the shaded region. The `Trial Number` `Session Number` effects are *FLMM* coefficient estimate plots.

We reasoned that if photobleaching caused the within-session decrease post-lick, indications of photobleaching would also be evident in other time-windows in the trial when the average signal was high (e.g., the pre-lick period). We assumed this would hold even if photobleaching depended on light intensity in a non-linear fashion (Serra and Terentjev, 2008), since we reasoned the relationship would still be monotonic. The signal–`Trial Number` association is, however, actually slightly positive pre-lick (albeit non-significant), indicating that the signal does not decrease on average within-session pre-lick. Moreover, if the degree of photobleaching scales with signal magnitude, then we would expect to see a negative signal–`Trial Number` association only during the [0,0.5] sec time-window when the mean signal is higher than it was during the pre-lick period (since the pre-lick period does not exhibit any negative signal–`Trial Number` correlation). Instead, the negative correlation is evident in a time-window when the mean signal has returned to close to zero [0, 2.5] sec. In Appendix Figure 11, we show the same analyses conducted on each session separately. *FLMM* estimates a large positive `Trial Number` effect pre-lick and post-lick on session 4, despite the fact that the average signal was higher than on sessions 1-3 when the `Trial Number` is negative. Since there are sessions on which it is possible to detect positive `Trial Number` effects, we reasoned that photobleaching would not have occluded any true DA increases within-session. Finally, we repeated the above analyses while adjusting for lick frequency and found that controlling for various behavioral engagement summaries did not impact the `Trial Number` effects.

In Appendix Figure 14, we conducted a similar analysis but aligned the signal to reward-delivery. If photobleaching were the main cause of the within-session reductions described above, one might expect the `Trial Number` coefficient to be most negative when the average signal is most positive. Instead, these analyses show that the within-session signal decrease (i.e., the negative `Trial Number` effect) and the average signal exhibit distinct temporal dynamics. For example, the peak average signal occurs around 0.75 sec post-reward, while the `Trial Number` coefficient is most negative around 1.25-1.5 sec. Moreover, while the average signal magnitude has returned to nearly 0 around 1.75 sec, the `Trial Number` coefficient remains significantly negative until at least 2.5 sec after reward-delivery.

bioRxiv preprint doi: https://doi.org/10.1101/2023.11.06.565896; this version posted October 19, 2024. The copyright holder for this preprint (which was not certified by peer review) is the author/funder. This article is a US Government work. It is not subject to copyright under 17 USC 105 and is also made available for use under a CC0 license.

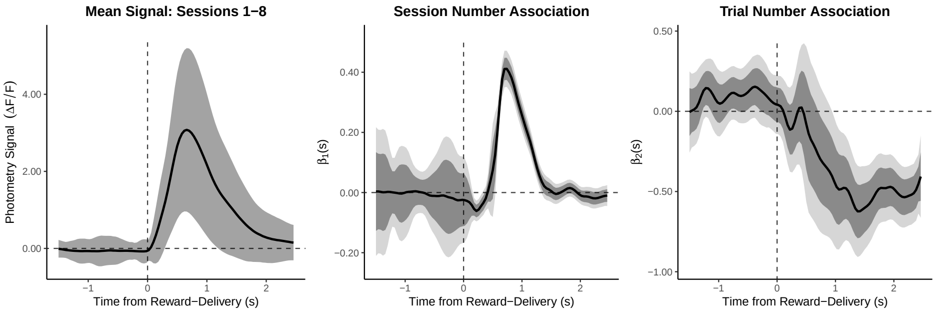

Figure 14: **Random `IRI` experiment aligned to reward-delivery: `Reward Number`–DA association analyzed as within-session (`Trial Number`) and between-session (`Session Number`) linear effects.** The average reward signal shows the average trace with standard error of the mean indicated by the shaded region. The `Trial Number` `Session Number` effects are *FLMM* coefficient estimate plots.

4.3.1 Photobleaching: Delay Length Experiment

We next sought to determine whether within-session decreases occurred in a different reward learning task collected from the same mice. We analyzed cue responses on the data from the Delay Length experiments (presented in main text Section 2.4). We used data from the final short-delay session because the animals were well trained on the Pavlovian task at that stage. During personal communications, Jeong et al. (2022) suggested these analyses because it provided an opportunity to analyze stabilized event-triggered DA responses in a different task from the same animals. Figure 15 shows the peak magnitude of the average signal at cue-onset (of the delay length data) is about 4.5 $\Delta F/F$ units (see the Intercept plot), which is about 15% *higher* than the peak magnitude of the average signal during the post-lick period of the experiment descried above (5 shows that the peak magnitude of the average signal was about 4 $\Delta F/F$ units). Because the signal is higher and photobleaching is thought to exert a greater effect on larger signals, one would expect photobleaching to have a *larger* effect in this experiment (i.e., a more negative `Trial Number` effect). However, Figure 15 shows that the within-session signal *increases* significantly over trials during the cue period (i.e., a positive `Trial Number` effect). Figure 15 also shows that the signal decreases within-session across trials at reward-delivery (3 sec after cue-onset) despite exhibiting a substantially lower average signal than during the Cue Period. This echos the within-session reductions observed around reward-consumption on the random `IRI` task above. We note additional analyses that adjusted for anticipatory licking and other indications of behavioral engagement did not noticeably impact the `Trial Number` effects. Analyses that pooled together multiple short-delay sessions yielded similar results. Taken together, these analyses of data from the same animals provide additional evidence against a photobleaching explanation as the main contributor for the within-session reduction described in Figure 5. One potential caveat is that there may be additional unmeasured factors (e.g., change in motivation) that increase across trials within a session, thereby occluding an effect of photobleaching. However, we reason that the parsimonious explanation in this case is that photobleaching is not a significant concern.

bioRxiv preprint doi: https://doi.org/10.1101/2023.11.06.565896; this version posted October 19, 2024. The copyright holder for this preprint (which was not certified by peer review) is the author/funder. This article is a US Government work. It is not subject to copyright under 17 USC 105 and is also made available for use under a CC0 license.

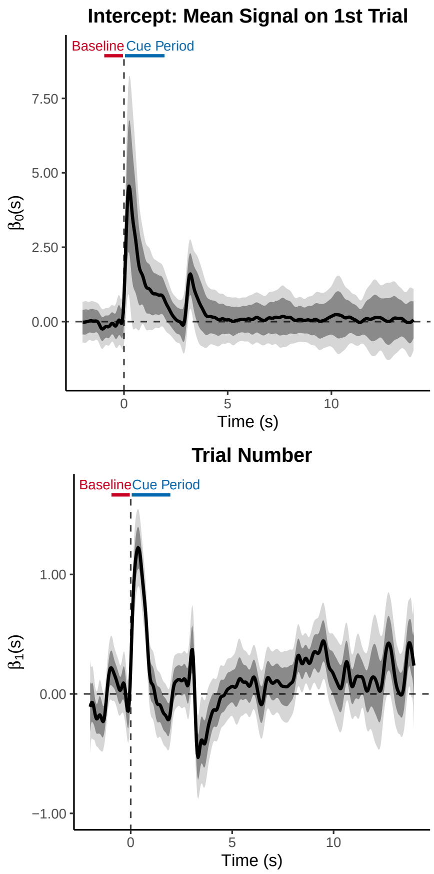

Figure 15: ***FLMM*** **identifies how the signal increases across trials during Cue Period and decreases across trials after reward-delivery (3 sec).**

4.3.2 Photobleaching: Background Reward Experiment

Next we show results from analyses suggested during personal communications with Jeong et al. (2022) to further rule out the possibility that the within-session decrease was a result of photobleaching. We specifically reanalyzed "Test 7" in Jeong et al. (2022) (original analysis results shown in Figure 4M-P of their manuscript). They described the experiment as follows:

> "To test whether the significant positive dopamine responses following extinction reflect a retrospective association between the cue and reward, we selectively reduced the retrospective association without reducing the prospective association. We maintained the fixed reward following the cue but added unpredictable rewards during the inter-trial interval. In this experiment, not all rewards are preceded by the cue (i.e., retrospective association is weak), but all cues are followed by reward (i.e., prospective association is high). ANCCR predicts a rapid drop in dopamine cue response whereas RPE predicts no change in cue response if TDRL only considers the cue-reward "trial period" (Test 7, fig. S10). The dopamine cue response remained significantly positive but decayed across trials faster than during extinction."

bioRxiv preprint doi: https://doi.org/10.1101/2023.11.06.565896; this version posted October 19, 2024. The copyright holder for this preprint (which was not certified by peer review) is the author/funder. This article is a US Government work. It is not subject to copyright under 17 USC 105 and is also made available for use under a CC0 license.

This experiment was like long-delay sessions in the Delay Length experiment (i.e., a CS+ followed by reward-delivery 9 sec later), but it also included rewards delivered randomly without any predictive stimuli during the inter-trial interval. These "background rewards" were similar to the reward-delivery schedule in the random `IRI` experiment presented in Figure 5 and therefore provide a critical point of comparison. Similar to our random `IRI` experiment analyses, we analyzed the same "reward period" time window aligned to the first lick after reward-delivery. We removed background reward "trials" that were too close to each other to avoid signal bleed-over from adjacent trials (e.g., trials with inter-reward intervals that were too short), and trials for which no licks occurred between two successive reward-deliveries (background or reward-predicted) to avoid "double-counting" a trial.

Figure 16 shows *FLMM* estimates a negative `Trial Number` effect. The magnitude of this negative coefficient is comparable to that seen in the random rewards experiments post-lick (i.e., after the first lick following reward-delivery). However, the within-session effect is most negative pre-lick, unlike what we observed in the random `IRI` task presented in Figure 5. It seems unlikely that this would be solely explained through satiation since the animals cannot be consuming the reward in the pre-lick period. Finally, we note additional analyses that adjust for lick frequency and other indications of behavioral engagement did not impact the `Trial Number` effects. Thus, while these results might be expected if photobleaching was causing the within-session signal reductions shown in Figure 5, we argue that the results suggest that it is unlikely that photobleaching is the sole contributor to these within-session reductions.

bioRxiv preprint doi: https://doi.org/10.1101/2023.11.06.565896; this version posted October 19, 2024. The copyright holder for this preprint (which was not certified by peer review) is the author/funder. This article is a US Government work. It is not subject to copyright under 17 USC 105 and is also made available for use under a CC0 license.

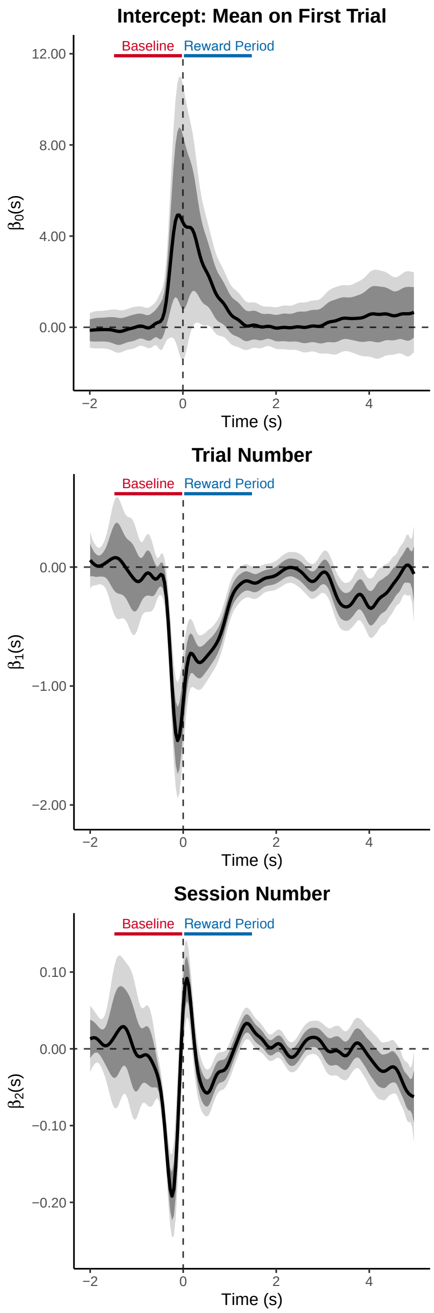

Figure 16: **Background Reward Experiment Analyses**

4.4 Using *FLMM* to test signal changes within- and across-trials

We show how *FLMM* enables hypothesis testing of signal changes within- and across-trials. In the experiment in Section "Tests 9 to 11" of Jeong et al. (2022), the authors test whether, across several sessions of Pavlovian learning, DA activity "backpropagates" from reward delivery (3 sec after cue-onset) to the presentation of reward-predictive cues.

bioRxiv preprint doi: https://doi.org/10.1101/2023.11.06.565896; this version posted October 19, 2024. The copyright holder for this preprint (which was not certified by peer review) is the author/funder. This article is a US Government work. It is not subject to copyright under 17 USC 105 and is also made available for use under a CC0 license.

They analyzed a summary measure defined as the difference between the average signal during pre-reward-delivery ("Late") and cue-onset ("Early") time-windows.

We tested the "backpropagation" question with a *FLMM* model with session binary indicators as covariates, similar to a functional repeated measures ANOVA. This yields estimates of mean signal changes, at each time-point, between pairs of sessions. We did not observe significant "Late" period changes, consistent with the authors' findings (Figure 17; see Appendix Figure 18 for individual-animal fits). This analysis likely cannot be used to definitively rule out the existence of the "backpropagation" phenomenon, but emphasizes how *FLMM* is well-suited to answer these types of questions. Nevertheless, we identified an additional effect that would be hard to find with summary measure analyses: the average (peak) size of cue-elicited DA, exhibited later in training, is similar to the degree that reward-delivery DA decreased. This is evident by comparing the symmetry between the magnitudes of peak increases during the "Early" period and peak decreases during the post–"Late" period on the last session (Figure 17D). This illustrates the capability in *FLMM* of directly testing effects visible in graphs, instead of having to perform hypothesis tests on a summary-of-summaries (e.g., a ratio or difference of AUCs).

bioRxiv preprint doi: https://doi.org/10.1101/2023.11.06.565896; this version posted October 19, 2024. The copyright holder for this preprint (which was not certified by peer review) is the author/funder. This article is a US Government work. It is not subject to copyright under 17 USC 105 and is also made available for use under a CC0 license.

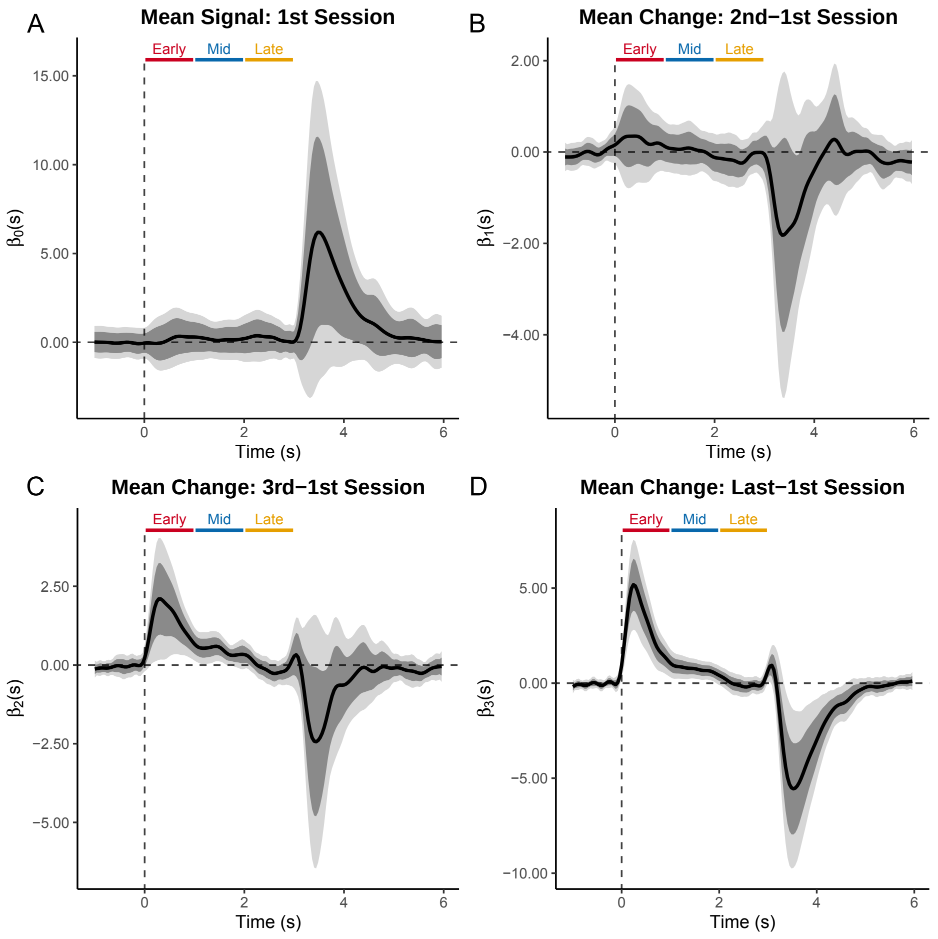

Figure 17: ***FLMM* identifies how the signal evolves across trial time-points, and how the temporal location of transients progresses across sessions in a statistically significant manner.** The panels show coefficient estimates from *FLMM* analyses of the "backpropagation" experiment. Panel (A) contains the intercept term plot corresponding to the average signal on the first session of training. The "Early" (0-1 sec), "Mid" (1-2 sec), and "Late" (2-3 sec) bars show the trial time-periods that the original authors used to calculate summary AUC measures. Trials are aligned to cue onset (cues lasted 2 sec) and rewards were delivered at 3 sec. Panels (B)-(D) show the coefficient estimates corresponding to the mean change in signal values from 2nd, 3rd or 4th sessions, respectively, *compared to the first session* (positive values indicate an increase from the first session). Plots are aligned to cue onset.

bioRxiv preprint doi: https://doi.org/10.1101/2023.11.06.565896; this version posted October 19, 2024. The copyright holder for this preprint (which was not certified by peer review) is the author/funder. This article is a US Government work. It is not subject to copyright under 17 USC 105 and is also made available for use under a CC0 license.

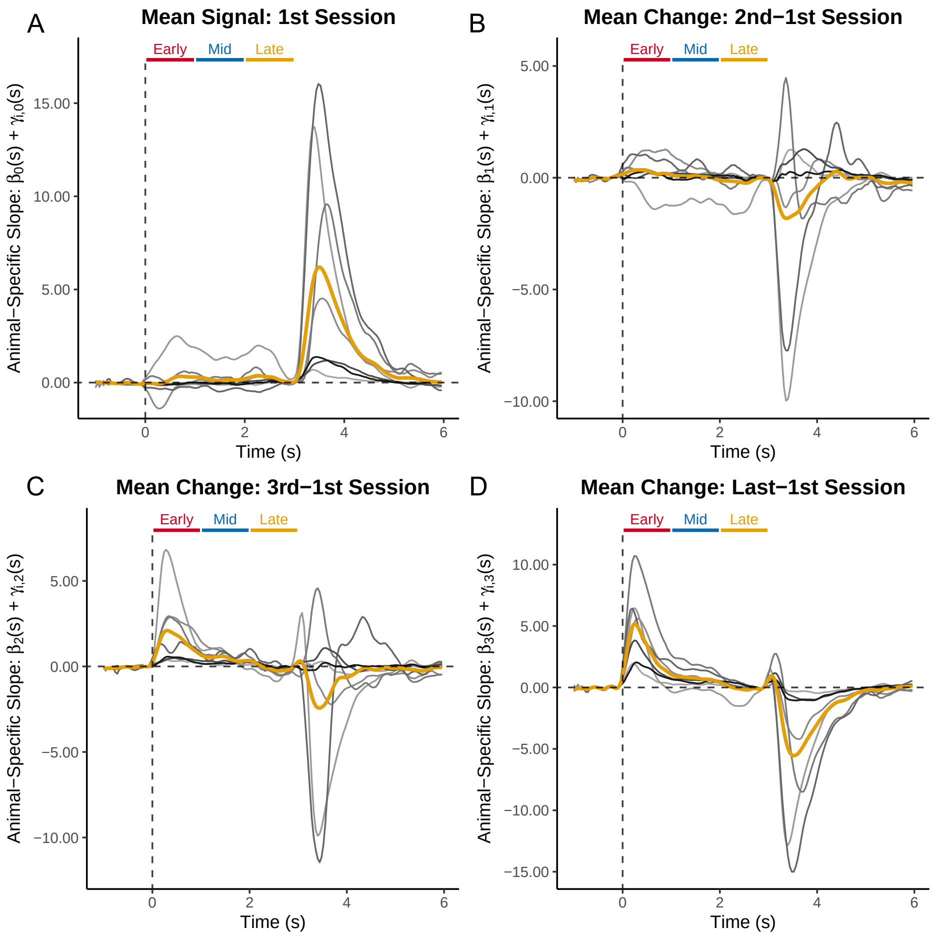

Figure 18: Individual-level coefficient estimates from *FLMM* analyses of "backpropagation" experiment: gold lines indicate the fixed-effect estimates and grey lines indicate animal-specific functional random-effect estimates (Best Linear Unbiased Predictor). Panel (A) contains the intercept term plot where the title provides an interpretation: the average signal on the first session of training. The "Early" (0-1 sec), "Mid" (1-2 sec), and "Late" (2-3 sec) bars show the trial time-periods that the original authors used to calculate summary AUC measures. Trials are aligned to cue onset and rewards were delivered at 3 sec. Panels (B)-(D) show the coefficient estimates which are interpreted as the change in mean signal from 2nd, 3rd or 4th sessions, respectively, compared to the first session (positive values indicate an increase from the first session). Plots are aligned to cue onset.

4.4.1 Reanalysis Methods: Using *FLMM* to test signal changes within- and across-trials

We reanalyzed data presented in the section "Tests 9-11 (Backpropagation within a trial)" of (Jeong et al., 2022). We sought to evaluate the author's hypothesis described in the following paragraph in the main text:

> "[in the] TDRL RPE account (...) dopamine responses drive value learning of the immediately preceding state. We tested three predictions of this central postulate that are each inconsistent with ANCCR. The first is that during the acquisition of trace conditioning, dopamine response systematically back-propagates from the moment immediately prior to reward to the cue onset

bioRxiv preprint doi: https://doi.org/10.1101/2023.11.06.565896; this version posted October 19, 2024. The copyright holder for this preprint (which was not certified by peer review) is the author/funder. This article is a US Government work. It is not subject to copyright under 17 USC 105 and is also made available for use under a CC0 license.

> (50) (Test 9, Fig. 6A). Unlike TDRL RPE, ANCCR does not make such a prediction since delay periods are not broken into states in ANCCR (...) Our observations were not consistent with a backpropagating bump of activity and were instead consistent with an increase in cue response over trials of learning (Fig. 6B) "

We analyzed data from sessions 1-3, and the final session for each animal, as these were the sessions where we noticed the greatest changes. We fit a random slope model using indicator variables of the 1st, 2nd, 3rd, and subject-specific final sessions as covariates. We discarded data from other sessions and thus the interpretation of the intercept is the mean signal on the first session. Our final random slope model was

$$\begin{aligned}\mathbb{E}[Y_{i,l}(s) \mid \mathbf{X}_{i,l}, \mathbf{Z}_{i,l}, \boldsymbol{\gamma}_i(s)] =& \beta_0(s) + \gamma_{0,i}(s) + \mathbb{1}(l=2)\left[\beta_1(s) + \gamma_{1,i}(s)\right] \\ &+ \mathbb{1}(l=3)\left[\beta_2(s) + \gamma_{2,i}(s)\right] + \mathbb{1}(l=\mathtt{S}_i)\left[\beta_3(s) + \gamma_{3,i}(s)\right].\end{aligned}$$

where l denotes the session number, $\mathtt{S}_i$ is subject i's final session (which can differ between animals), and $\mathbb{1}(l = s)$ is an indicator variable for session s. We show the code from our package to fit the above model (note that the `period` variable below is a factor variable):

```
model_fit = fui(photometry ~ period + (period | id), data=photometry_data)
```

5 Appendix: Simulations

5.1 Appendix: Simulation Scheme

As described in the main text, we simulated data from the model

$$Y_{i,j}(s) = \beta_0(s) + \gamma_{0,i}(s) + \mathtt{Delay}_{i,j}\left[\beta_1(s) + \gamma_{1,i}(s)\right] + \epsilon_{i,j}(s). \tag{12}$$

We take the simulated $\boldsymbol{\beta}(s)$ in 4.7 to be equal to the estimated coefficients from model 12 fit to the real data in (Jeong et al., 2022). To "simulate" the covariates (i.e., just the `Delay` indicator vector), we randomly draw a subset of animal IDs and concatenate all of their *observed* covariates from the (Jeong et al., 2022). That is for each simulation replicate, r, of sample size $n \in \{4, 5, ..., 8\}$, we randomly draw a sample of n animal subject IDs, denoted as n_r from the set $\{1, 2, ..., 7\}$ with uniform probability without replacement. For each animal ID in the sample, n_r, we concatenate the covariates (i.e., the design matrix) of the corresponding subjects in the *observed* data to be the "simulated" covariates. That is, the design matrix $\mathbb{X}_r$ for simulation replicate r, is the row concatenation of each $\mathbb{X}_i$ for $i \in n_r$.

5.2 Appendix: Additional Simulation Results

To explore how analyzing summary measures can drown-out effects, we compared method performances on the same analyses across different cue period lengths (2 sec, 2.5 sec, or 3 sec from cue onset), which we visualize in Figure 19A. These relatively small adjustments substantially influenced estimation performance (see Figure 19B), and statistical power (see Figures 19D and 20. The pointwise 95% CI coverage (see Figure 19C) of the

bioRxiv preprint doi: https://doi.org/10.1101/2023.11.06.565896; this version posted October 19, 2024. The copyright holder for this preprint (which was not certified by peer review) is the author/funder. This article is a US Government work. It is not subject to copyright under 17 USC 105 and is also made available for use under a CC0 license.

summary measure approaches (t-test and LMM) is also very sensitive to the specified length of the cue period because they analyze a summary that averages over a time-window that contains heterogeneous effects. However, *FLMM* and *Perm* CI coverages (averaged over the cue period) remain stable to differing time-window lengths, because they evaluate each time-point. Notably, *Perm* exhibits low coverage at smaller sample sizes and the t-test yields poor coverage in all settings tested, likely because of the animal-to-animal variability in the signal magnitude and Delay Length change effect.[8] Finally, Appendix Figure 21 shows *FLMM* fits in around 10 seconds for datasets with about 800 trials (pooled across animals). Taken together, these data-driven simulations demonstrate that at low sample sizes, and in the presence of individual-differences, the *FLMM* consistently 1) achieves roughly nominal *pointwise* and *joint* coverage, 2) improves statistical power, and 3) can be fit quickly.

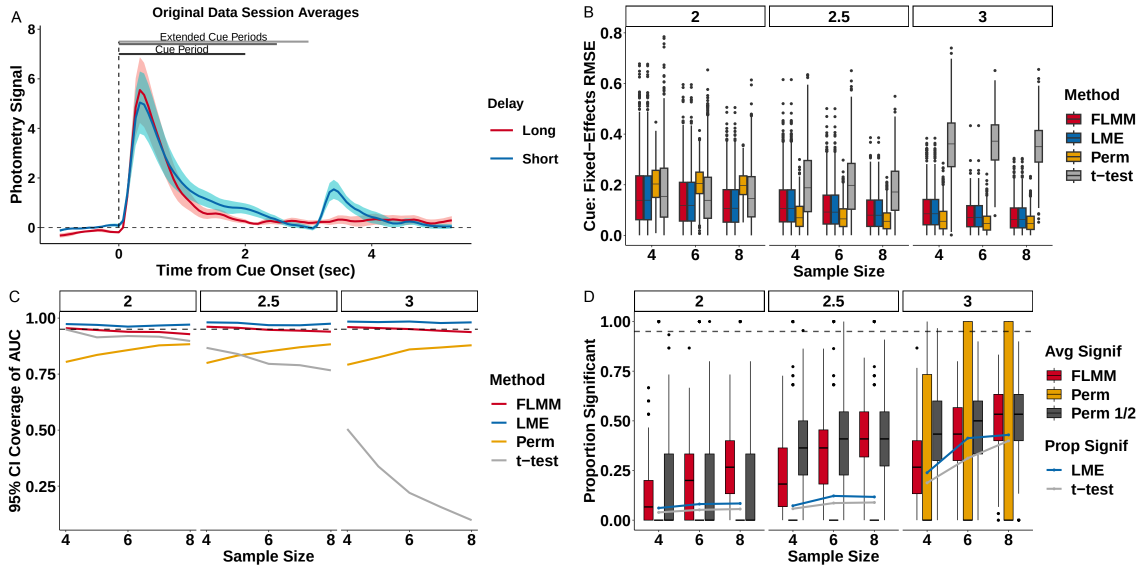

Figure 19: Summary measure analyses are highly sensitive to minor changes in the summary time-window. (A) Average short/long-delay data. Bars show cue period length used in (2 sec) and "extended" delays analyzed in additional simulations. (B) Estimation error (RMSE), $\frac{1}{\sqrt{R}}\left\|\bar{\boldsymbol{\beta}}_1(\boldsymbol{s}) - \bar{\hat{\boldsymbol{\beta}}}_1(\boldsymbol{s})\right\|_2$ where $\bar{\boldsymbol{\beta}}_1(\boldsymbol{s}) = \frac{1}{|\mathcal{S}|}\sum_{s\in\mathcal{S}}\beta_k(s)$, associated with mean difference during the cue periods (panels) and n on the x-axis. Lower numbers indicate more accurate estimates. (C) pointwise 95% CI coverage associated with mean difference during cue period (panels) and n on the x-axis. Higher values indicate better CI coverage. (D) Statistical power during cue period. The LMM and t-test were fit on the signal averaged over the cue period and thus each simulation replicate yields a single indicator of CI inclusion or statistical significance, which we represent with a line plot. For other methods, estimates are provided at each time-point and performance is averaged across the time-points. We summarize these simulation replicate-specific averages with a boxplot.

Here we define the estimation error (RMSE) of statistical methods for the average difference in photometry signal amplitude during the cue period as a function of how long that cue period was (2 sec, 2.5 sec, 3 sec). The error was defined as $\frac{1}{\sqrt{R}}\left\|\bar{\boldsymbol{\beta}}_1(\boldsymbol{s}) - \bar{\hat{\boldsymbol{\beta}}}_1(\boldsymbol{s})\right\|_2$

[8]The data were simulated from a LMM that specifies independence between observations conditional on random-effects. Since the t-test does not include random-effects, it may not achieve the nominal coverage because it relies on a different conditional independence assumption.

bioRxiv preprint doi: https://doi.org/10.1101/2023.11.06.565896; this version posted October 19, 2024. The copyright holder for this preprint (which was not certified by peer review) is the author/funder. This article is a US Government work. It is not subject to copyright under 17 USC 105 and is also made available for use under a CC0 license.

where $\bar{\boldsymbol{\beta}}_1(\boldsymbol{s}) = \frac{1}{|\mathcal{S}|}\sum_{s\in\mathcal{S}}\beta_1(s)$. That is, the average of the coefficients for covariate k across time-points s in a fixed interval (indexed by $\mathcal{S}$). Since the model only contains a single binary covariate, we can compare the average of functional coefficients for the slope parameter in a *FLMM* model with the slope coefficient estimate in a scalar LMM applied on the outcome: $\bar{Y}_{i,j} = \frac{1}{|\mathcal{S}|}\sum_{s\in\mathcal{S}} Y_{i,j}(s)$ and a paried-samples t-test using the outcome: $\bar{Y}_i = \frac{1}{|\mathcal{J}_i|}\frac{1}{|\mathcal{S}|}\sum_{s\in\mathcal{S}}\sum_{j\in\mathcal{J}_i} Y_{i,j}(s)$, where $\mathcal{J}_i$ is the set of trials observed for subject i.

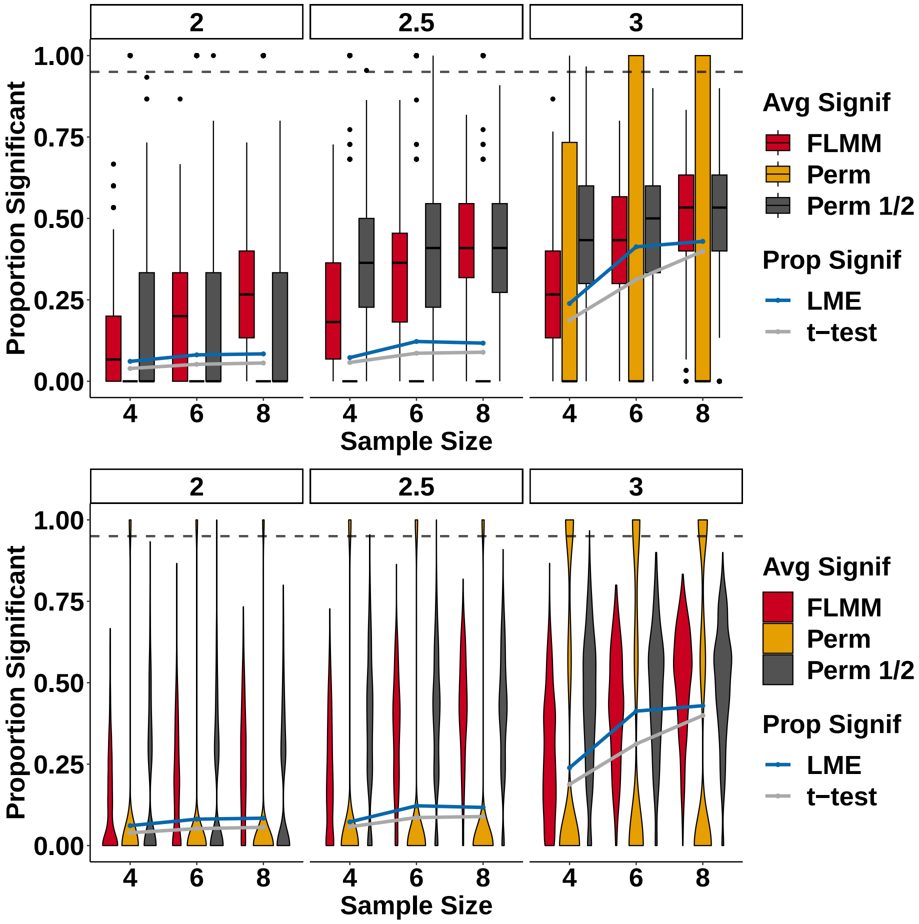

Figure 20: Statistical power associated with mean difference defining the cue period as 2 sec, 2.5 sec, and 3 sec (panels) and sample sizes (numbers of animals) on the x-axis. The two panels presents the same data in either violin or boxplot forms. Higher numbers indicate better power. For *FLMM* and *Perm*, power is averaged across the time-points in the cue period whereas the others assess the power using the average signal (across the cue period) as the outcome. Since each simulation replicate takes the proportion of significant time-points in the cue period for *FLMM* and *Perm*, these are presented as boxplots (or violin plots), whereas the rest are simply presented as the proportion of simulation replicates that identified the mean signal during the cue as statistically significant (either 0 or 1 for each replicate).

bioRxiv preprint doi: https://doi.org/10.1101/2023.11.06.565896; this version posted October 19, 2024. The copyright holder for this preprint (which was not certified by peer review) is the author/funder. This article is a US Government work. It is not subject to copyright under 17 USC 105 and is also made available for use under a CC0 license.

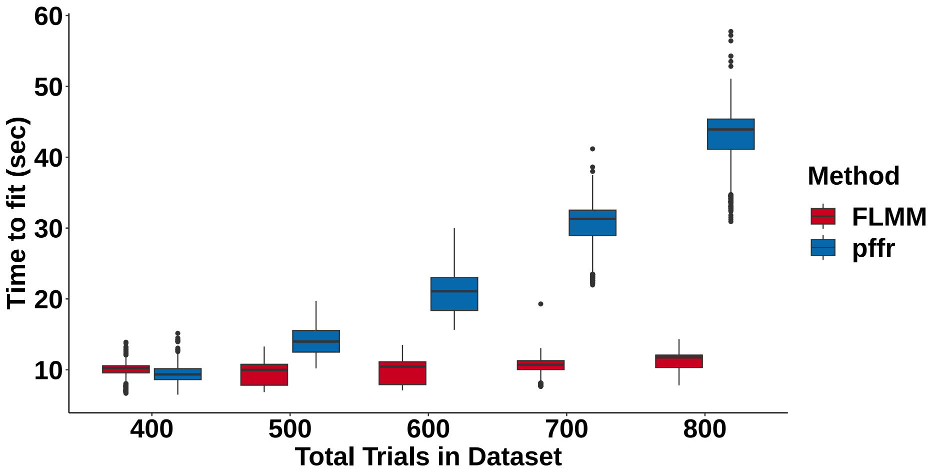

Figure 21: Time to fit *FLMM* fit with our software (using a closed-form variance calculation) on simulated data (each datapoint represents one replicate). `pffr` shows the time to fit the functional linear mixed model (with the same model specification) with the `pffr()` function in the `refund` package. Number of animals in simulations shown in plots ranges from 4-8 (i.e., $n = trials/100$).

bioRxiv preprint doi: https://doi.org/10.1101/2023.11.06.565896; this version posted October 19, 2024. The copyright holder for this preprint (which was not certified by peer review) is the author/funder. This article is a US Government work. It is not subject to copyright under 17 USC 105 and is also made available for use under a CC0 license.

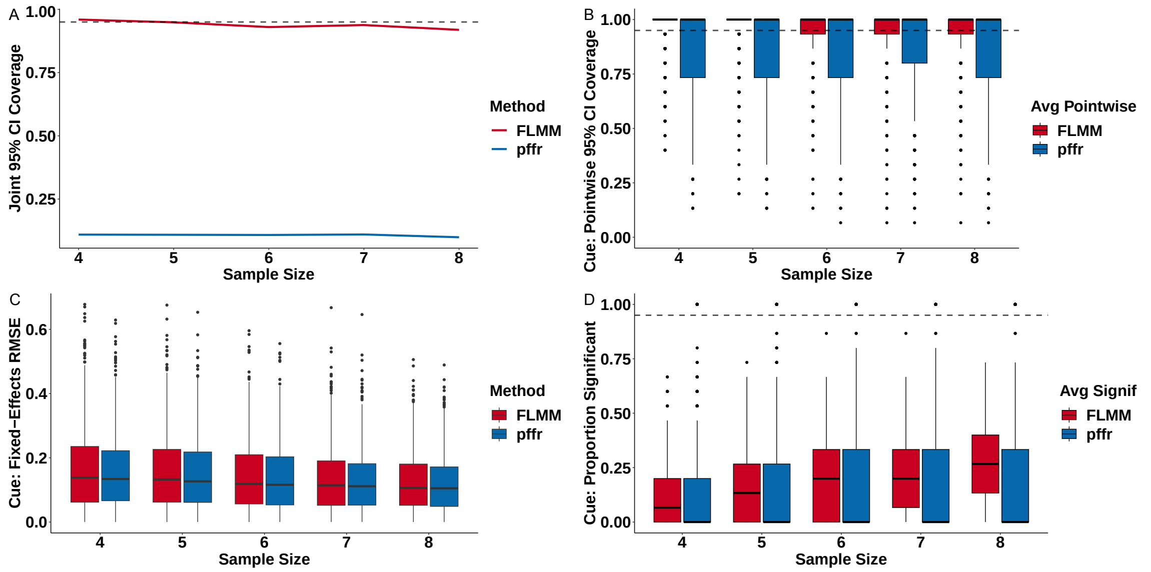

Figure 22: *FLMM* fit with our software achieves comparable or superior performance to the functional linear mixed model (with the same model specification) fit with the `pffr()` function in the `refund` package. (A) *FLMM* achieves *joint* 95% CI coverage at roughly the nominal level. `pffr` does not provide *joint* 95% CIs and thus the *pointwise* 95% CIs that it does provide achieve low *joint* coverage. (B) *FLMM* achieves *pointwise* 95% CI coverage at or above the nominal level. The *pointwise* 95% CI coverage of `pffr` is close to but below the nominal level. *pointwise* 95% CI coverage associated with mean difference during cue period (panels) and n on the x-axis. Higher values indicate better CI coverage. (C) *FLMM* and `pffr` exhibit comparable fixed-effects estimation performance. Estimation error (RMSE), $\frac{1}{\sqrt{R}}\left\|\bar{\boldsymbol{\beta}}_1(\boldsymbol{s}) - \bar{\bar{\boldsymbol{\beta}}}_1(\boldsymbol{s})\right\|_2$ where $\bar{\boldsymbol{\beta}}_1(\boldsymbol{s}) = \frac{1}{|\mathcal{S}|}\sum_{s\in\mathcal{S}}\beta_k(s)$, associated with mean difference during the cue periods (panels) and n on the x-axis. Lower numbers indicate more accurate estimates. (D) *FLMM* exhibits superior statistical power compared to `pffr` during the cue period. (B)-(D) Since estimates are provided at each time-point for both methods, pointwise performance is averaged across the time-points. We summarize these simulation replicate-specific averages as one point in a boxplot.

6 Appendix: Additional Reanalyses

We include analyses here conducted on a second recent article Coddington et al. (2023); Dudman (2023) proposing a new reinforcement learning model for the role of mesolimbic dopamine in learning.

6.1 Appendix: Additional Reanalyses Results

6.1.1 Functional methods allow for testing how signal "dynamics" early in training predict behavior later in training

We next analyze data from a second paper focused on the role of mesolimbic DA in reward learning (Coddington et al., 2023). We first examine how between-animal differences in behavior correlate with average nucleus accumbens dopamine neuron calcium

bioRxiv preprint doi: https://doi.org/10.1101/2023.11.06.565896; this version posted October 19, 2024. The copyright holder for this preprint (which was not certified by peer review) is the author/funder. This article is a US Government work. It is not subject to copyright under 17 USC 105 and is also made available for use under a CC0 license.

changes (NAc–DA).[9] In this experiment, mice were exposed to a 0.5 sec stimulus, followed by reward 1 sec after cue-offset. The authors identified significant correlations between average Reward period NAc–DA (trial-averaged on *early* training sessions) with measures of average behavior (trial-averaged on *late* sessions).

We fit two analogous univariate models with the average behavioral measures as the covariate.[10]

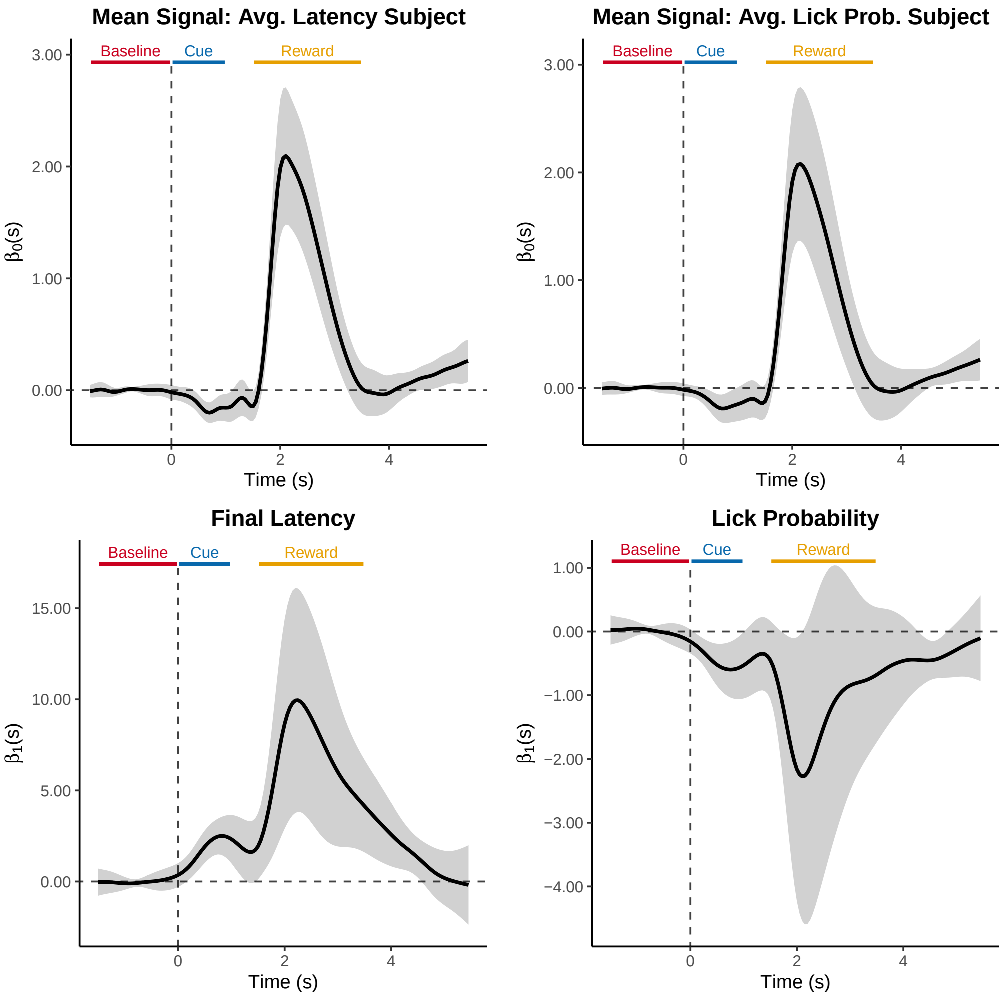

Figure 23: Coefficient estimates from *FLMM* analyses of `Final Latency` and `Lick Probability` models. The top row contains intercept term plots where the title provides interpretation of the intercept: the average NAc-DA signal on trials when the covariates in the model are at their average value. The bottom row shows the coefficient estimate plot of the covariate in the model. (Left) Association between average NAc-DA (averaged over first 100 trials) and latency to lick `Final Latency` (averaged over trials 700-800). (Right) Association between average NAc-DA (averaged over first 100 trials) lick probability (averaged over trials 700-800).

[9]DAT-Cre::ai32 transgenic mice were injected with a Cre-dependent jRCaMP1b virus across the ventral midbrain enabling the measurement of calcium dynamics in mesolimbic DA cells specifically.

[10]Cases without repeated observations on the same animal are equivalent to a *functional* linear regression (without mixed effects).

bioRxiv preprint doi: https://doi.org/10.1101/2023.11.06.565896; this version posted October 19, 2024. The copyright holder for this preprint (which was not certified by peer review) is the author/funder. This article is a US Government work. It is not subject to copyright under 17 USC 105 and is also made available for use under a CC0 license.

Our results reveal details missed in the summary measure analyses. Consistent with the author's findings, we found Reward period NAc–DA was associated with `Final Latency`. However, our analyses also revealed significant associations during the Cue period, an effect hard to see on average traces: while the average signal increases substantially during the Reward period, it exhibits comparatively little change during the Cue period. This demonstrates the difficulty of constructing summary measures: the time-windows when the signal is associated with covariates may not align with time-periods when the average signal exhibits noticeable changes. Remarkably, the association sign differs from the direction of average signal change: `Final Latency` is *positively* associated with NAc-DA during both Cue and Reward periods, yet the mean signal *decreases* during the Cue Period and *increases* during the Reward period.

6.1.2 *FLMM* provides test of how signal differences *between* trial-types change *across* training

Our final example demonstrates how questions that might otherwise require analysis of summaries of summary measures (e.g., ratios of average AUCs), can be precisely specified in a functional model so as to provide greater detail. Specifically, the authors sought to investigate how the difference in NAc-DA signals between trials with (Lick+) and without (Lick-) preparatory licking changed with learning. They reported a significant correlation between reward collection latency (`Final Latency`) and a "ratio of NAc–DA reward signals [AUCs] on Lick- vs Lick+ trials." We fit an *FLMM* model with an interaction between `Final Latency` and `Lick State` (a Lick-/Lick+ indicator). The interaction term provides a hypothesis test of their question at *each trial time-point.* The longest portion of joint significance occurs *between* Cue and Reward periods. Our analysis confirms the authors' results and adds detail obscured by standard methods: the interaction does not reach joint statistical significance until a couple seconds after reward-delivery, well-beyond the time when the average signal has fallen from its peak (shown by the intercept). This example highlights the challenge with constructing an adequate summary measure for this analysis: the period of clearest effect of the `Lick State` is before the average signal is largest. This may explain why the authors did not construct a summary measure during the interval between the Cue and Reward periods, when the `Lick State` effect was strongest. In sum, this example demonstrates how summary-of-summary measure analyses can be translated into simple *FLMM* regressions that provide greater detail about the time-course and magnitude of the effects throughout the trial.

bioRxiv preprint doi: https://doi.org/10.1101/2023.11.06.565896; this version posted October 19, 2024. The copyright holder for this preprint (which was not certified by peer review) is the author/funder. This article is a US Government work. It is not subject to copyright under 17 USC 105 and is also made available for use under a CC0 license.

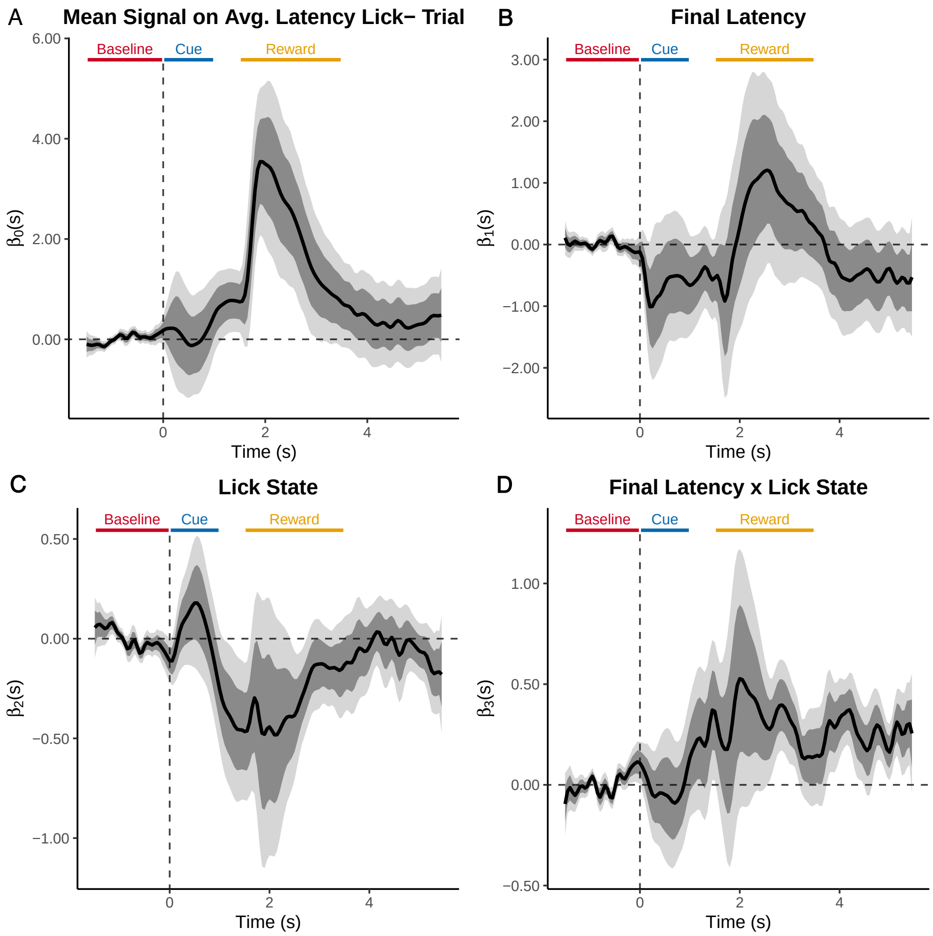

Figure 24: Coefficient estimates from a single *FLMM* analysis of `Final Latency` × `Lick State` interaction model (including main effects). A simple *FLMM* model enables characterization of how the association between photometry signals and behavioral responding (`Final Latency`) differs between conditions (Lick+/Lick-) at each time-point in the trial. Panel (A) contains the intercept term plot where the title provides an interpretation: the average photometry signal on Lick- trials for animals that exhibit average `Final Latency` values. Panels (B)-(D) show the three covariates of main effects and interaction of `Lick State` and `Final Latency`. The `Final Latency` functional coefficient is interpreted as the effect of `Final Latency` on Lick- trials. The `Lick State` main effect is interpreted as the difference in average NAc–DA between Lick+ and Lick- trials for an animal with an average `Final Latency` value. The interaction is interpreted as a difference in differences: during the Reward period, a 1 standard deviation increase in average `Final Latency` is associated with pointwise significantly higher NAc–DA signals on Lick+ than on Lick- trials (with a portion of joint significance) during most of the Reward period.

6.2 Appendix: Additional Reanalyses Methods

6.2.1 Methods for Appendix Section 6.1.1

This paper measured photometry signals in which DAT-Cre::ai32 transgenic mice were injected with a Cre-dependent jRCaMP1b virus across the ventral midbrain enabling the measurement of calcium dynamics in mesolimbic DA cells specifically. We sought to conduct an analysis based upon the following quote:

bioRxiv preprint doi: https://doi.org/10.1101/2023.11.06.565896; this version posted October 19, 2024. The copyright holder for this preprint (which was not certified by peer review) is the author/funder. This article is a US Government work. It is not subject to copyright under 17 USC 105 and is also made available for use under a CC0 license.

> "Unexpectedly, initial NAc–DA reward signals were negatively correlated with the amount of preparatory behaviour at the end of training (NAc–DA reward trials 1-100 versus preparatory index trials 700-800, r=-0.85, P=0.004), as well as the speed of reward collection (NAc-DA reward trials 1-100 versus reward collection latency trials 700-800, r=0.81, P=0.008)."

Preparatory licking and latency-to-lick were used as indicators of learning.

Since there is no way to meaningfully pair the neural activity of one trial with behavior on a separate trial, analyzing trial-level data is not appropriate and we therefore modeled the *average* photometry signal (averaged across trials 1-100) as a function of *average* behavior (averaged across trials 700-800). We removed trials with latencies over 1 second as they constituted behavioral outliers comprising less than 1% of trials.

To conduct a functional regression most analogous to the Pearson correlations the authors conducted, we fit the univariate linear regression models for final latency, $\texttt{FL}_i$,

$$\mathbb{E}[\overline{Y}_i(s) \,|\, \mathbf{X}_i] = \beta_0(s) + \texttt{FL}_i \beta_1(s).$$

Similarly, the we fit the lick probability, $\texttt{LP}_i$, functional regression model,

$$\mathbb{E}[\overline{Y}_i(s) \,|\, \mathbf{X}_i] = \beta_0(s) + \texttt{LP}_i \beta_1(s).$$

These models were fit with the `R` package `refund` with the `fosr`() function (where fosr abbreviates function-on-scalar regression used for a functional outcome and scalar covariate). Note that this package function only provides *pointwise* 95% CIs and thus the plots in the main text only have one shade of grey for the 95% CIs. Thus the interpretation for this analysis is confined to a *pointwise* interpretation.

6.2.2 Methods for Appendix Section 6.1.2

We sought to conduct an analysis based upon the following quote: "[the proposed] scheme also predicts that [mesolimbic DA] reward signals should reflect the evolution of reward collection policy across learning...Indeed,...mouse data exhibited differential reward responses on trials with ('Lick+') or without ('Lick-') preparatory licking as learning progressed" (Coddington et al., 2023). The authors reported (see the Figure 8d caption (Coddington et al., 2023)) a significant Pearson correlation between the final reward collection latency (`Final Latency`) and a "ratio of NAc–DA reward signals on Lick- vs Lick+ trials." They constructed this measure by first summarizing the reward-period NAc-DA (with AUC), averaged across Lick+ and Lick- trials separately, and then calculating a ratio for each animal. Similar to the analysis we presented in the previous section, the authors compared behavior on one set of trials to dopamine activity in a separate set of trials. Because there is no way to meaningfully pair the neural activity of one trial with behavior on a separate trial, we analyzed the *average* photometry signal (Lick+/Lick- separately averaged over the trials specified in the paper) as a function of *average* behavior. Specifically, we modeled the trial-averaged signal denoted as $\overline{Y}_{i,v}(s)$ for animal i and lick state v. The signal was trial-averaged separately across Lick+ and Lick- trials. We both indicate lick state with the subscript v on $\overline{Y}_{i,v}(s)$ and also as a covariate in the model where $\texttt{LS}_{i,v} = 1$ for Lick+ trials and $\texttt{LS}_{i,v} = 0$ for Lick- trials.

bioRxiv preprint doi: https://doi.org/10.1101/2023.11.06.565896; this version posted October 19, 2024. The copyright holder for this preprint (which was not certified by peer review) is the author/funder. This article is a US Government work. It is not subject to copyright under 17 USC 105 and is also made available for use under a CC0 license.

We denote the final latency below as $\texttt{FL}_i$. We fit the random slope *FLMM* model,

$$\mathbb{E}[\overline{Y}_{i,v}(s) \,|\, \mathbf{X}_{i,v}, \mathbf{Z}_i, \boldsymbol{\gamma}_i(s)] = \beta_0(s) + \gamma_{0,i}(s) + \texttt{FL}_i \left[\beta_1(s) + \gamma_{1,i}(s)\right] + \texttt{LS}_{i,v}\beta_2(s) + \texttt{FL}_i \times \texttt{LS}_{i,v}\beta_3(s).$$

Given that the outcome was trial-averaged, the number of observations of the outcome was only twice the number of animals (i.e., one observation of the functional outcome for Lick+ and one for Lick-). We were thus limited in the number of random-effects we could include and still retain an identifiable model. For that reason, our model did not include a random intercept. As for all other analyses in our manuscript, we compared multiple candidate models that we specified based upon the authors analyses. We then selected the model with the best AIC/BIC model fit criteria.

We normalized the (average) `Final Latency` variable (across trials) to have mean 0 and unit variance. The intercept therefore shows that mean NAc–DA activity for a mouse with average `Final Latency` on Lick- trials is *jointly* significantly elevated during portions of the Reward period. The `Final Latency` functional coefficient is interpreted as the effect of `Final Latency` on Lick- trials and is only briefly pointwise significant in the middle of the Reward period. The `Lick State` main effect is interpreted as the difference in average NAc–DA between Lick+ and Lick- trials for an animal with an average `Final Latency` value. The interaction is interpreted as a difference in differences: during the Reward period, a 1 standard deviation increase in average `Final Latency` is associated with pointwise significantly higher NAc–DA signals on Lick+ than on Lick- trials (with a portion of joint significance) during most of the Reward period.
